# Orchard netting impacts on biodiversity leading to cascading effects at the ecosystem level

**DOI:** 10.1002/brv.70186

**Published:** 2026-05-19

**Authors:** Corrado Alessandrini, Karan Sethi, Emanuela Granata, Ekaterina Mogilnaia, Valeria Vitangeli, Giovanni Zanfei, Chiara Fedrigotti, Paolo Biella, Dino Scaravelli, Luigi Marchesi, Péter Batáry, Diego Rubolini, Paolo Pedrini, Mattia Brambilla

**Affiliations:** ^1^ Department of Environmental Science and Policy University of Milan Via Celoria, 26 Milan I‐20133 Italy; ^2^ Faunistics and Wildlife Conservation, Department of Agriculture, Ecotrophology, and Landscape Development Anhalt University of Applied Sciences Bernburg DE‐06406 Germany; ^3^ Department of Animal Ecology & Systemics Justus Liebig University Gießen Heinrich‐Buff‐Ring 26‐32 Giessen DE‐35392 Germany; ^4^ Biologia della Conservazione, Museo delle Scienze di Trento – MUSE Corso del Lavoro e della Scienza 3 Trento I‐38122 Italy; ^5^ Dipartimento Scienze Storiche e dell'Antichità – Dissgea University of Padua Via del Vescovado, 30 Padova I‐35141 Italy; ^6^ Department of Biotechnology and Biosciences, ZooPlantLab University of Milano‐Bicocca Milan I‐20126 Italy; ^7^ Dipartimento di Scienze Biologiche, Geologiche ed Ambientali – BiGeA University of Bologna Via Zamboni, 33 Bologna I‐40126 Italy; ^8^ MTA–HUN‐REN Centre for Ecological Research, Institute of Ecology and Botany ‘Lendület’ Landscape and Conservation Ecology Alkotmány U. 2‐4 Vácrátót Hungary

**Keywords:** agroecology, ecosystem services, permanent crops, pest control, pollination, pome fruits, protection covers, protective nets, species‐specific environmental filtering, vineyards

## Abstract

Agriculture must ensure food production without further compromising the ecosystem functions upon which it depends. Agricultural practices should therefore avoid harming farmland biodiversity, especially of taxa that supply the key ecosystem services (e.g. pollination, pest control and nutrient uptake) that ultimately support crop production. Orchards are among the largest permanent plantations worldwide and are increasingly characterised by the spread of plastic nets used to protect fruits/nuts from either abiotic (anti‐hail, anti‐rain, shade nets) or biotic (exclusion nets) hazards. Despite having received little attention to date, these nets may impact natural communities, acting both as physical barriers and as drivers of habitat changes to which biota must respond. Species‐level responses to netting depend on the organism's ability to enter the netted environment and successfully exploit available resources. Net‐mediated ecological filtering and plastic behavioural responses may alter species interactions, leading to cascading ecological impacts that may create species‐poorer ‘netted communities’ with simplified ecological networks. Such changes may erode biological control potential, other ecosystem functions, and overall system stability. We conducted a systematic review on the effects of protection nets on biota, and reported novel empirical evidence on anti‐hail nets' impacts on communities of orchard‐dwelling birds, flower‐visiting insects, and rodents. In total, we identified 48 studies from the literature, however this literature was strongly biased towards apple orchards, western countries, and pest taxa. Net deployment was highly effective in deterring target pest species, in some cases regardless of their original function, as even weather‐protection nets limited pest populations. Side effects on non‐target taxa were also often reported, such as decreases in pollinators and natural enemies, and/or increases in secondary pests or microbial diseases. However, most assessments largely disregarded non‐pest taxa and the broader ecological consequences of netting. The few studies that addressed the effects of nets at the guild/community level, including our empirical study, confirmed that orchard netting resulted in species‐poor assemblages, with possible ecosystem‐level consequences. We propose that future assessments should pay more attention to the indirect effects of netting on non‐target taxa, and on the supply of crop‐supporting ecosystem services mediated by wild species occurring in agroecosystems. Due to the trade‐offs between these services and net‐mediated crop protection, integrated alternatives should be tested to improve the environmental sustainability of food production and biodiversity conservation in farmed landscapes.

## I. INTRODUCTION

Agroecosystems largely depend on biodiversity for crucial functions such as biological pest control, pollination and soil services, which provide irreplaceable and economically relevant support to agricultural production (Buzhdygan & Petermann, 2023; Dainese *et al*., 2019). Yet, the current state of agricultural intensification poses a major threat to biota worldwide (Dudley & Alexander, 2017; Flohre *et al*., 2011). There is therefore a compelling urgency to prevent further loss of farmland biodiversity by identifying sustainable farming practices that ensure both resilient food production and the functioning of (agro)ecosystems.

Permanent crops cover 190 million ha globally (~12% of all croplands) and have expanded by 40% since 2001 (especially in Western Africa and South‐Eastern Asia; FAO, 2024). The largest share of permanent crops is represented by orchards, i.e. woody cultivations dedicated to fruit production (Simon *et al*., 2010). Temperate fruit crops (e.g. apples, peaches) largely predominate, but orchards may also include nut‐bearing trees (e.g. oil palms), vineyards and tropical shade crops (like cocoa, mango and coffee), as they all share similar structures, management and functions. In orchards, fruit trees are planted in stands of variable densities, largely depending on crop species and management intensity: high‐density row‐like configurations are now progressively replacing more traditional ones characterised by larger and sparser trees.

In recent decades, orchards have been subject to contrasting pressures. On the one hand, they have been pushed towards agricultural intensification to improve fruit quality and yields, and to be competitive on global markets. On the other hand, there has been growing demand to meet sustainability goals by reducing pesticides and synthetic inputs and by promoting more sustainable farming practices (Demestihas *et al*., 2017). As a consequence, an increasing number of studies have focused on the effects of orchard management on biodiversity (reviewed in van der Meer *et al*., 2020). Such approaches leverage fundamental ecological processes such as predator–prey and plant–insect interactions to identify sustainable ways to maximise ecosystem services (e.g. pollination, pest control) and minimise disservices (e.g. fruit damage; see e.g. Colloff, Lindsay & Cook, 2013; Daelemans *et al*., 2023; Garratt *et al*., 2023). Thanks to permanent cover, higher vegetation biomass and increased structural complexity, orchards can host richer biological communities and more complex food webs than most annual crops, increasing the delivery of biodiversity‐mediated ecosystem services that support crop production (Price *et al*., 1980; Shackelford *et al*., 2013). Hence, these systems may represent ideal ‘laboratories’ for developing biodiversity‐mediated sustainability measures (Martinez‐Nuñez *et al*., 2025). Moreover, orchards have also been identified as important suppliers of other key ecosystem services such as carbon sequestration and water regulation (Demestihas *et al*., 2017).

**Fig. 1:**
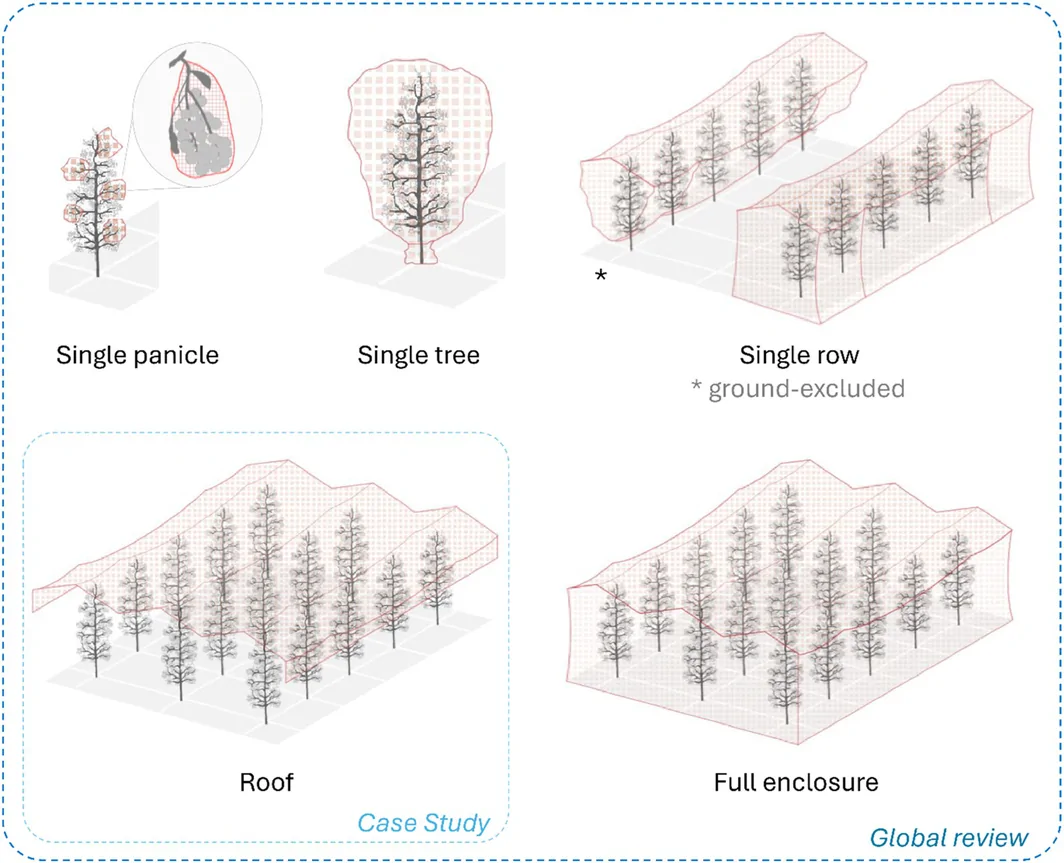
Possible configurations of protection nets in orchards. Depending on their function, nets are usually arranged in different configurations: nets against weather hazards (e.g. anti‐hail or shade nets) are usually roof or single‐row nets, while exclusion nets are more often full enclosures, single‐row, single‐tree or single‐panicle nets. The present global review investigated all net configurations, while the case study focused on roof (anti‐hail) nets. See Fig. S1 for photographic examples. Artwork by Alejandra León.

Among the plethora of modern agricultural practices, protection nets have been increasingly used in orchards at all latitudes since the late 20^th^ century. By acting as physical barriers over the crop, they are intended to optimise crop production by protecting the plants from (*i*) abiotic factors, i.e. excessive sunlight radiation and meteorological hazards (wind, rain, hail, snow and frost), or (*ii*) biotic pests, such as insects, vertebrates and microbial diseases (Castellano *et al*., 2008; Manja & Aoun, 2019). Nets are usually made of plastic (high‐density polyethylene, HDPE) and have structures and mesh sizes specific for their function (e.g. anti‐hail, anti‐insect, photo‐selective, anti‐rain; Castellano *et al*., 2008; see also Appendix S1). Protective nets can cover the entire orchard (termed ‘roof’ when open on the sides, or ‘full enclosure’ when both roof and sides are netted), single rows, single trees, or even single panicles (Fig. 1, see also Fig. S1). Usually, anti‐pest nets (named ‘exclusion nets’) more tightly adhere to the canopy, sometimes also excluding the ground beneath the trees (termed ‘complete exclusion nets’ in Chouinard, Firlej & Cormier, 2016), while anti‐hail or photo‐selective nets usually cover larger extents and are more loosely adhered to the trees. Depending on the timing of exposure to the threat, nets can be activated seasonally (e.g. during the rainy season for anti‐rain nets, or during fruiting for exclusion nets), or permanently (Elsysy *et al*., 2019; Fornasiero *et al*., 2023).

Nets usage also comes with unintended effects, primarily involving the physical conditions induced under the nets. Extensive literature has explored this topic from an agronomic perspective (Bosco *et al*., 2017; Dussi *et al*., 2005; Iglesias & Alegre, 2006; Lodolini *et al*., 2018; Volz, Tustin & Ferguson, 1996). Most common effects include a reduction in air flow and wind speed, and an increase in relative humidity, which are all strongly mediated by the net configuration and its mesh size (Manja & Aoun, 2019). These net‐induced changes can be either advantageous, like lower irrigation costs due to reduced water evaporation under anti‐hail nets (McCaskill *et al*., 2016), or detrimental, like reduced ripening in apples grown under shade nets (Willsea *et al*., 2023).

While the effects of orchard netting on the physical environment, plant physiology and fruit production have been extensively reviewed (Castellano *et al*., 2008; Mahmood *et al*., 2018; Mditshwa, Magwaza & Tesfay, 2019; Mupambi *et al*., 2018; Rigden, 2008), no assessment systematically reviewed net effects on the biotic components of the orchard agroecosystem, like wild plants and animal communities. So far, only Chouinard *et al*. (2016), Manja & Aoun (2019) and Vuković *et al*. (2022) reviewed net effects on pest and disease biota, though from an agronomic perspective and overlooking the totality of orchard‐dwelling biodiversity. Yet, understanding net‐mediated changes on agroecosystem biodiversity may be relevant not only from a conservation perspective, but also for ensuring the delivery of crucial ecosystem services, mediated by these organisms.

With this study, we aim to fill this knowledge gap by (*i*) reviewing the effect of orchard netting on biodiversity at the global scale and (*ii*) presenting a case study on the responses of three illustrative taxa (birds, flower‐visiting insects, and rodents) to a specific net type (anti‐hail nets) in Italian apple orchards. Upon such combined evidence, we propose a conceptual framework on the ecological mechanisms involved with (and affected by) orchard netting, and highlight the possible trade‐offs associated with the use of protection nets.

We preliminarily identify the main ecosystem‐level features which orchard‐dwelling biota should cope with, after a netting system is introduced. Despite the great variability of environmental conditions determined by the orchard itself and the net characteristics (function, structure and mesh size), five features may be highlighted. Firstly, organisms, like many animals or animal‐pollinated plants, likely experience higher *isolation* in netted orchards. The net covers (and the abiotic changes induced) affect above‐ground flow of organic and inorganic matter (especially in the case of finer mesh sizes and complete exclusion configurations). Secondly, after the disturbance due to net deployment (often limited to very short installation or activation time), the increased weather protection under nets changes weather‐related *disturbance regimes* (Castellano *et al*., 2008). When exclusion nets replace pesticide applications, their adoption can also reduce management‐related disturbances (Lefebvre *et al*., 2016). Thirdly, netted orchards have altered *structural complexity*, through the addition of artificial structures (poles, wires and the net itself) and constrained tree growth and shape (Rigden, 2008). Fourthly, environmental perturbations are moderated when the nets are active but are reestablished once nets are deactivated. Hence orchards seasonally/temporally covered by nets likely experience altered patterns of (year‐round) *temporal fluctuations*. Finally, for organisms that manage to thrive within complete exclusion netting systems (either single‐tree, single‐row or full enclosures), the netted environment is characterised by a *reduced spatial extent*, which may severely limit habitat connectivity and the amount of resources available therein. A glossary of some of the terms used in this review can be found in Table 1.

**Table 1:** A glossary of terms used in this review.

Orchard	A permanent plantation of any fruit‐ or nut‐bearing trees whose production is oriented towards fresh markets and fruit‐derived products. We apply no latitudinal boundary, hence adopting a wider definition than in Simon *et al*. (2010) and van der Meer *et al*. (2020) that only focused on temperate crops, e.g. apples, pears, peaches, citrus. We thus include under ‘orchards’ also inter‐tropical crops (e.g. coffee, bananas, avocados, mangos) and vineyards, which often show comparable conditions as the temperate fruit crops.
Protection/protective net	A covering structure arranged over or around the crop to protect it from potential damages exerted by both abiotic (e.g. excessive solar radiation, rain, wind, hail, or snow) and biotic factors (e.g. animal pests or microbial diseases). Less‐preferred synonyms: (protective) cover, shield, enclosure, cage, screen, tunnel.
Agroecosystem	An area devoted to agricultural production. Its ecosystemic flows are largely controlled by human‐regulated inputs (e.g. fertilisers, biocides, and water) and outputs (e.g. crop harvest, weed removal).
Ecosystem service/disservice	Conditions, functions or processes through which a natural ecosystem, and the species that make them up, sustain or benefit (services) or hamper (disservices) human life (Daily, 1997). In agroecosystems, these relate particularly to crop production and the processes on which it depends, such as soil retention and fertility, water regulation, pollination, pest control, and nutrient uptake (services), or herbivory and disease infections (disservices).
Crop disease	A microbial taxon belonging to viruses, bacteria, or fungi, whose occurrence/development within the orchard causes damage to any tree part (rooting system, trunk, branches, leaves, flowers or fruits) and negatively impacts crop production (either in terms of tree's health or fruit yield/quality).
Crop pest	An animal taxon (mostly, arthropod or vertebrate) whose occurrence within the orchard results in reduced crop health or production.
Non‐pest	Any taxon whose occurrence within the orchard does not negatively affect crop production. Among this category, some taxa (namely, pest controllers) might reduce the impact of diseases, weeds, or pests (e.g. by feeding on them) so that they might exert an indirect positive effect on the crop.
Agri‐functional group	A categorisation of the orchard‐dwelling biota according to their ecosystemic role, with reference to their effect on the crop (i.e. the prefix ‘agri‐‘). We distinguish four broad groups: microbial diseases, pests, weeds, and non‐pests. The latter can be further split between (positive) disease−/weed−/pest‐controllers, (negative) pest‐alleys and disease‐vectors, and a generic ‘other’ (including all those organisms which have no direct effect either on the crop, on the diseases nor pests).

## II. METHODS

### (1) Systematic review

To assess the effects of protection nets on orchard‐dwelling biodiversity, we conducted a systematic review of the literature using two databases, ISI Web of Science (WOS) and Scopus. We followed the guidance provided by Foo *et al*. (2021) for systematic reviews in ecology. After some initial scoping with preliminary search terms, we performed the literature search by using the following search string:

((protective OR protection OR exclusion OR enclosure OR hail OR rain OR frost OR wind OR snow OR shade OR selective OR photoselective OR proof) NEAR/0 (net* OR screen* OR cage$ OR enclos*)) **AND** (biodiversity OR bird* OR mammal* OR insect$ OR reptile* OR amphibian* OR invertebrate* OR vertebrate* OR pest$ OR pollinat* OR fauna* OR ((ground OR wild) NEAR/0 (flora OR flower* OR plant* OR vegetation*))) **AND** (orchard* OR vineyard* OR agro‐forest* OR grove$ OR ((fruit* OR nut OR tree OR permanent OR perennial) NEAR/1 (crop* OR plantation* OR culture*))) **AND** (impact* OR effect*).

Search date: 19^th^ March 2025; WOS database: ‘Web of Science Core Collection (SCI‐EXPANDED)’; the string was searched on ‘Article Title, Abstract, Keywords’ for both WOS and Scopus. In Scopus, ‘Pre/x’ was used instead of ‘NEAR/x’. We applied no temporal restrictions, including all papers published before the search date.

#### (a) Inclusion and exclusion criteria

We combined the results from both platforms, removed the duplicates, and reviewed title, abstracts and – when relevant – the full text. We included studies with any quantitative field assessment of a direct impact of (any type of) protective net on any biological parameter (e.g. occurrence, abundance, behaviour, fitness, incidence on crop) related to any non‐crop taxon within or near the orchards (of any fruit or nut). We excluded studies (*i*) investigating nets' impact only on abiotic factors, crop physiology or crop phenology; (*ii*) carried out in laboratory settings; and (*iii*) that used experimental netting to test the effect of a taxon on the crop (e.g. exclosure experiments, as in Maas *et al*., 2019). Eventually, we screened references of the retrieved literature to include additional relevant studies that may have been missed by our search.

### (2) Case study

We carried out a field study in the apple orchards of the Non Valley (in the north of Italy, province of Trento), where roof anti‐hail nets are widely adopted to protect the crop from hailstorms. This wide glacial valley is currently one of the primary areas of apple production in Europe, accounting for ~17% of total Italian apple production (Italy being the second largest European apple producer; European Commission, 2025). In the valley, more than 96% of the orchards are planted with apples, which largely adopt integrated pest management (IPM), while <5% adopts organic farming. Apple trees are tightly set up in rows (mean intra‐row × inter‐row distance between neighbouring trees: 0.89 × 3.2 m), and arranged in particularly small stands, defined as *parcels* (i.e. a portion of the orchard that shares the same agronomic, structural, and management features; mean ± SE of parcel size: 1,391 ± 8 m^2^). In the whole valley, apple orchards cover ~6,414 ha, of which ~999 ha are covered by roof anti‐hail nets (~15.6% of the cultivated area; mesh size 3 × 7 mm). Anti‐hail nets are scattered throughout the valley (Fig. S2), and are activated from the withering of flowers (mid‐May) until the harvest period (September/October).

Within this system, we investigated birds, flower‐visiting insects, and rodents in 63 sites (hereafter plots), each consisting in a 100‐m buffer around a 200 m‐long linear transect, for a total of 7.14 ha each. The plots were scattered over the valley to encompass the whole environmental variability of the study area (Fig. S3) and included tens of single apple parcels (mean ± SE: 32 ± 1 parcels per transect; 80.3 ± 1.1% total orchard cover per transect). Fieldwork took place from late April to early September 2023, and each plot was visited several times throughout the season (Table S1). Overall, we collected parcel‐wise data on the abundance and diversity of (*i*) birds and (*ii*) flower‐visiting insects, and (*iii*) on the abundance of rodent burrows (used as a proxy for rodent densities; Šálek *et al*., 2025). We also collected structural, management and environmental variables in each sampled parcel within the plots (Table S2); among these variables, we recorded the occurrence of anti‐hail nets and their state: active (i.e. open, spread over the parcel) or inactive (i.e. closed, wrapped up; Fig. S2C). To increase sample size, rodent burrows were also sampled in June 2025. A full description of the sampling activities is provided in Appendix S2.

#### (a) Statistical analysis

We assessed the effect of active anti‐hail nets separately on birds, insects and rodents by means of generalised linear mixed‐effect models (GLMMs). As response variables we used (*i*) the abundance of birds found in each apple parcel overlapping with the plots at each visit; (*ii*) the species richness of birds found throughout the three visits in each apple parcel overlapping with the plots; (*iii*) the overall number and (*iv*) the ‘taxon richness’ of flower‐visiting insects in each sampling sub‐plot; and (*v*) the abundance of rodent burrows in each sampled parcel. Since we identified flower‐visiting insects at broad taxonomic levels (from genus to order; see Appendix S2), we calculated their diversity by counting the number of taxa present, hence we will use ‘taxon richness’ rather than ‘species richness’. To account for the non‐independent (stratified) nature of the datasets, we tested plot identity as a random factor and retained it when supported (i.e. lower Akaike's information criterion, AIC); for bird abundances, which were surveyed three times during the season and had multiple records per parcel, the parcel ID was also included as another random factor (see Table S3); for rodents, the year of the sampling (either 2023 or 2025) was also included as a random factor. Response variables were modelled using a Poisson error distribution, shifting to a negative binomial one in case of significant overdispersion (*P* < 0.05).

To estimate the effect of nets while accounting for other variables relevant to the ecology of each taxon (i.e. topography, orchard management and distance from other land‐cover classes), we built a global model with the ‘hail‐net’ factor (0: anti‐hail net absent or inactive; 1: anti‐hail net active) and all other relevant environmental variables (Table S2), controlling for multicollinearity (Variance Inflation Factors, VIF <4; James *et al*., 2021). We evaluated the statistical support for the ‘hail‐net’ factor in terms of significance (*P* < 0.05) of the estimated effect size. We inspected all models for outliers, zero‐inflation, uniformity and autocorrelation of residuals, by means of dedicated functions (Appendix S3).

## III. RESULTS

### (1) Review

We highlight semantically contrasting terminology with regard to operating nets, with contradictory use of ‘open’ and ‘closed’ nets between exclusion (‘closed’ when enclosing the crop; Fornasiero *et al*., 2023) and roof nets (‘open’ when covering the crop; Schluchter, Späth & Buchleither, 2022). To avoid this ambiguity, we use henceforth, and suggest to use in the future, the term ‘active’ to refer to nets in a working set up (e.g. roof anti‐hail nets spread over the crop, or exclusion nets tightly enclosing the canopies), and ‘inactive’ for nets in a non‐working set up (i.e. wrapped up).

The database search returned 106 results from Scopus and 82 from Web of Science. After excluding duplicate records, 124 studies were screened, of which 37 met our inclusion criteria. Following reference screening, eleven additional studies were identified and included, resulting in a final sample of 48 reviewed studies (Fig. S4 for the PRISMA diagram; see also Replication Data for the full list of studies). Together, these studies reported 134 quantified effects of netting on biodiversity.

**Fig. 2:**
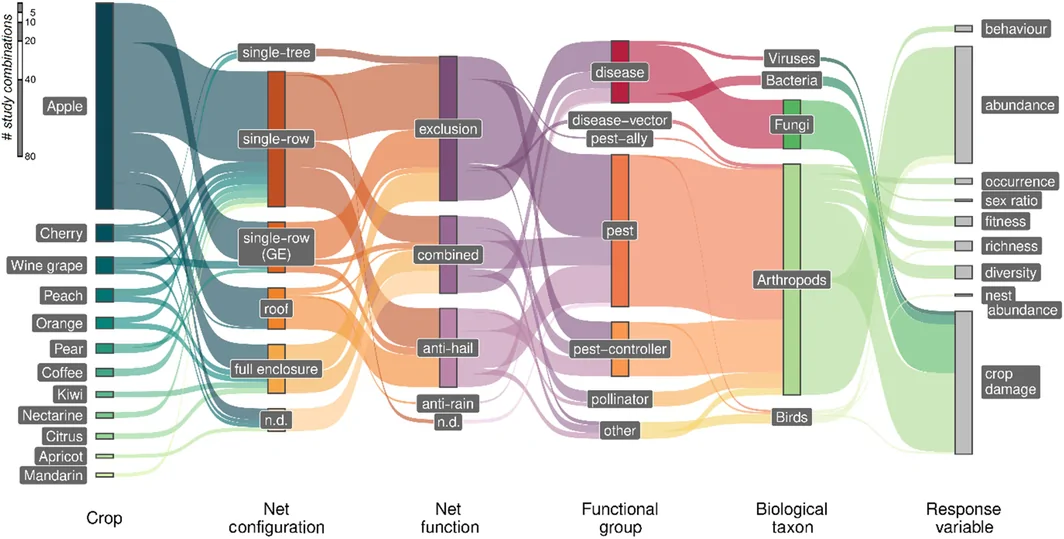
Network chart with the most relevant features found in the 48 reviewed studies on the effects of orchard netting on biodiversity, namely (from left to right): crop species under study, net configuration and net function tested, functional groups and taxa studied, and which biological parameter was evaluated as response variable. The height of each vertical block is proportional to the number of studies that assessed that level (reference on the top left). The width of the shaded paths denotes the number of studies that share the two features connected by each path. Note the uneven coverage across crops (apples overrepresented), functional groups (very few studies on pollinators) and taxa (complete lack of studies on plants and any vertebrates but birds). GE stands for ground‐excluded. Figure made with R‐packages ‘ggsankey’ and ‘ggplot2’.

**Fig. 3:**
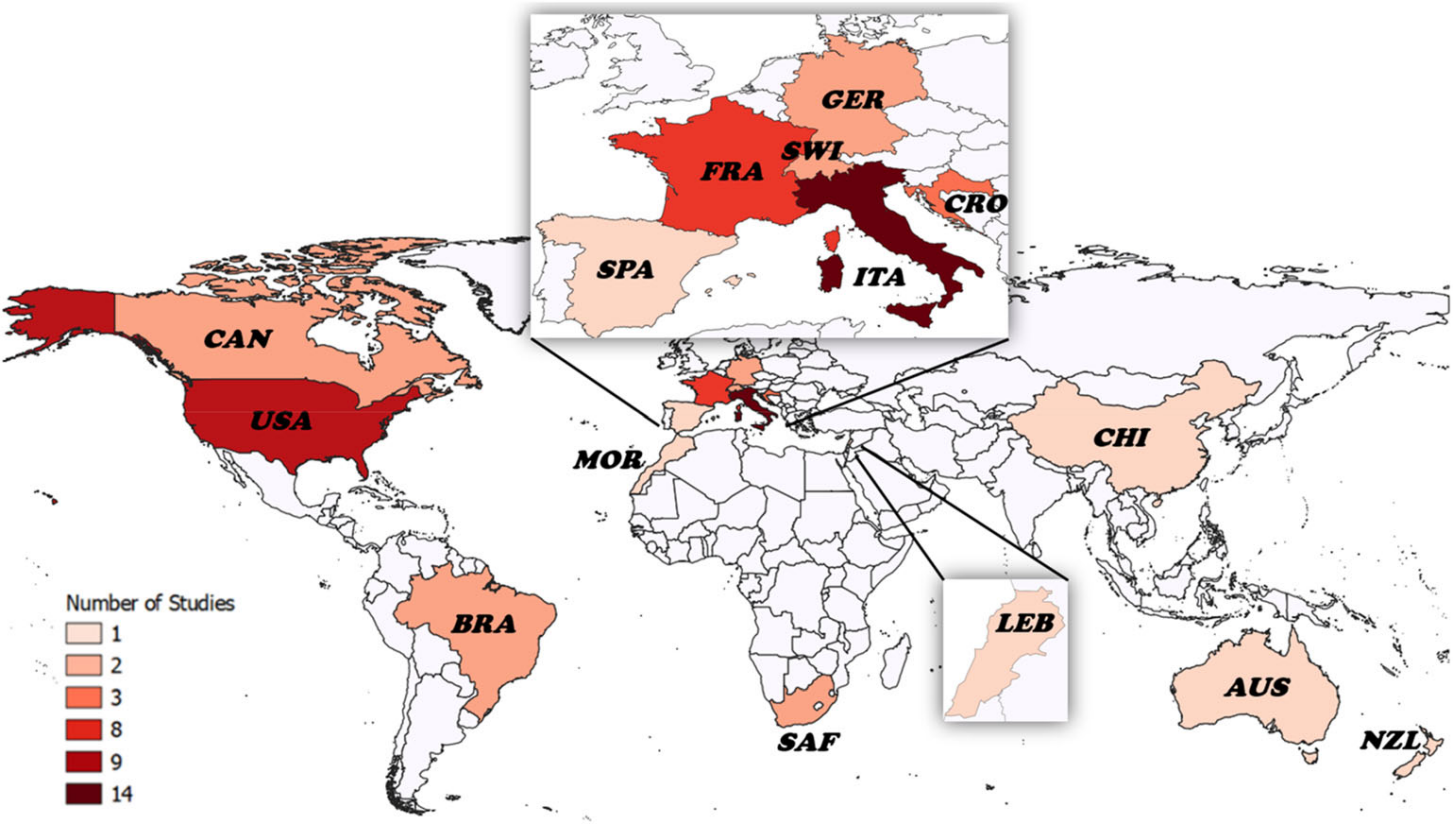
Global map on the origin of the reviewed literature (*N* = 48 studies) about the effects of orchard netting on non‐crop biodiversity. The darker the colour, the more studies from that country. Most of the studies were carried out in Europe and Northern America, while very few came from the inter‐tropical region. Country abbreviations (from West to East): CAN = Canada; USA = United States of America; BRA = Brazil; MOR = Morocco; SPA = Spain; FRA = France; SWI = Switzerland; GER = Germany; ITA = Italy; CRO = Croatia; SAF = South Africa; LEB = Lebanon; CHI = China; AUS = Australia; NZL = New Zealand.

Within the reviewed literature (*N* = 48 studies), the functional types of nets under study were mainly exclusion nets (*N* = 22) and anti‐hail nets (*N* = 16), while other studies tested combinations of either exclusion or anti‐hail nets with other net types (e.g. anti‐rain or photo‐selective nets; *N* = 12). Single‐panicle and single‐tree nets were the least studied net configurations (Fig. 2; no study investigated single‐panicle nets). The investigated taxa were arthropods (*N* = 40), fungi, bacteria, or viruses (hereafter, ‘microbial taxa’ for simplicity; *N* = 20 studies) and birds (*N* = 5); no studies investigated other vertebrates (amphibians, reptiles or mammals) or any wild plant species. Reviewed studies covered 12 crop species, but especially apples (*N* = 35; Fig. 2), and were mainly carried out in Europe and Northern America (Fig. 3). More than 40% of the studies (*N* = 21) were performed in experimental (i.e. non‐commercial) orchards. Twelve studies (25%) were carried out in organic orchards. Forty studies (83%) explicitly reported the mesh size of studied nets, with exclusion and combined nets having generally finer meshes than anti‐hail nets (Fig. S5).

The experimental design was almost invariably a treatment‐control approach, where a netted orchard plot (the treatment) was compared with an unnetted one (the control). Some of them also included additional treatments for comparison, like pesticide applications (e.g. Boutry *et al*., 2022) or different net types (Kelderer *et al*., 2018), mesh sizes (Charlot & Weydert, 2017), net colours (Bogo *et al*., 2012a,b) or net deployment timing (Stander *et al*., 2019). In general, the main focus was on the abundance of the studied taxon; at times, due to the difficulty in sampling (e.g. for microbial taxa) or due to the purely agronomic interest of the study, only nets' effect on the crop was quantified instead (e.g. infestation or fruit damage rates). In some cases, other parameters like birds' nest‐site selection or bees' flower visitation rates were assessed. For studies investigating entire guilds (e.g. birds or arboreal spiders), community indices were also at times assessed (Fig. 2). A more detailed report is presented in Appendix S4 (see also Fig. S6).

#### (a) Effects on pests and diseases

Most studies (77%; *N* = 37) investigated a taxon considered to be a crop pest or a microbial disease. Half of these studies investigated exclusion nets (*N* = 19), while the rest investigated the effect of other net types (e.g. anti‐hail, anti‐rain or combined nets) for the same purposes of disease or pest control.

Overall nets exhibited strong pest‐control capacity, since 24 out of the 25 studies that reported *P*‐values (or a similar metric of statistical support) documented a significant reduction in the abundance of at least one pest taxon. In some studies, no single pest specimen was found inside exclusion nets, despite their occurrence in unnetted controls (Lloyd *et al*., 2005; Marshall & Beers, 2022). Pest‐induced damages also decreased thanks to net protection (e.g. Sauphanor *et al*., 2012). Conversely, disease‐control through netting was somewhat weaker, with a significant reduction in disease occurrence (or damages) reported in just 5 out of 13 studies.

**Fig. 4:**
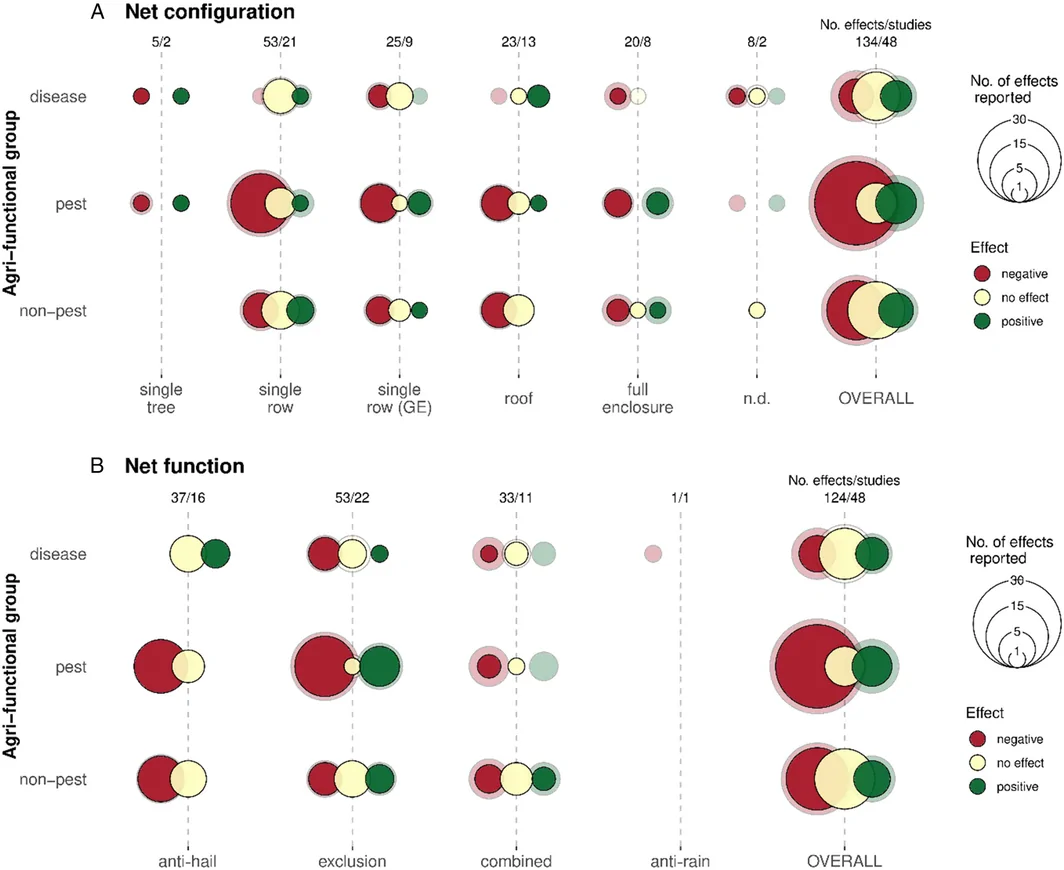
The effect of orchard netting on biodiversity as reported in literature (48 reviewed studies), distinguishing biota by their agri‐functional role (namely, disease, pest or non‐pest) and nets by their (A) structural configuration and (B) function. The overall effect exerted by any net on each group is presented on the right (‘OVERALL’ column). The size of each circle is proportional to the number of effects found in literature. Circles in bold colours represent the effects whose significance was statistically tested, while paler circles also include effects that were not tested for statistical significance. The total number of effects and studies is reported above each net type. Combined nets include two or more net types (e.g. anti‐hail, exclusion, anti‐rain, and/or photo‐selective nets). Nets exert a largely negative effect on biota (red circles bigger than yellow and green ones; especially on pests, but also on non‐pests); some net types are more prone to unintended increases of biota (e.g. full enclosures with pests; anti‐hail nets with diseases). GE = ground excluded.

In nine of these studies (24%), nets also led to a significant increase in microbial diseases (Bogo *et al*., 2012a,b; Lueje, Jácome & Servia, 2024) or insect pest species (Chouinard *et al*., 2023; Marsberg *et al*., 2024; Marshall & Beers, 2022, 2021; Fig. 4).

#### (b) Effects on non‐pest taxa

Twenty‐two studies (46%) assessed the effects of nets on non‐pest organisms, focusing especially on arthropod natural enemies (*N* = 17), like insect parasitoids, predatory mites, or spiders. Similarly to the effect found for pest taxa, nets generally reduced the abundance of non‐pest organisms. Out of 18 studies that reported test statistics, nets exerted a significant negative impact on non‐pest taxa in 12 studies, no significant impacts in 10 studies, and a positive impact in four studies (Fig. 4).

Only three studies focused on pollinator taxa: honey bees in Evans *et al*. (2019), wild pollinators in Meissle *et al*. (2023), and flower‐visiting insects – including honey bees – in Granata *et al*. (2025); all found a negative effect of nets on their abundance, flower visitation rates, and/or colony fitness. When tested on (non‐pest) birds (*N* = 4, all in apple orchards from Southern Europe or Northern Africa), nets always exerted a negative effect, on bird breeding densities (Brambilla *et al*., 2015; Brambilla, Martino & Pedrini, 2013), habitat use (Bouvier, Boivin & Lavigne, 2022), and nest‐site selection (Mansouri *et al*., 2020).

Finally, 24 studies (50%) investigated more than one taxon of the same ‘agri‐functional group’ (microbial disease, pest, pest‐controller/pollinator, or other). One fourth (*N* = 6) found significant but different (positive *versus* negative) effects within such groups, which means differential changes in intra‐guild and/or community composition under the nets.

### (2) Case study

According to the full models of each taxon, active anti‐hail nets in the apple orchards of the Non Valley showed a significant negative effect on the abundance and diversity of both birds and flower‐visiting insects, while showing a significant positive effect on the abundance of rodent burrows (Table 2; see Fig. S7 for all the effects included in the full models). This means that orchard netting favours a pest group – rodents – while hampering the occurrence of birds and flower‐visiting insects, i.e. the potential providers of pest control and pollination services.

**Table 2:** The effect of anti‐hail netting on the three taxa investigated in the case study, in the intensive apple orchards of the Non Valley (Trentino, Italy). Regression coefficients (*β*), adjusted standard errors, and 95% Confidence Intervals (CI) of the modelled effect of active nets are reported. Sample size, the marginal (without the random factor contribution) and conditional (with the random factor effect) *R* ^2^ of each model are also provided. For model specifications, see Table S3. Models marked with an asterisk have been worked out with parcel area (log‐transformed) as offset, while the reported *R* ^2^ refers to a model with ‘log(parcel_area)’ as a fixed effect rather than as the offset (to facilitate *R* ^2^ computation).

Taxon	Response	Sample size	*β* estimate	Adjusted standard error	*z* value	*P*	95% CI	*R* ^2^ (Marginal/Conditional)
Birds	Abundance	2310	−0.302	0.090	−3.36	<0.001	−0.48:−0.13	0.383/0.584*
	Richness	770	−0.212	0.078	−2.70	0.007	−0.37:−0.06	0.428/0.443*
Flower‐visiting insects	Abundance	907	−0.431	0.186	−2.32	0.020	−0.80:−0.07	0.894/0.904
	Richness	907	−0.366	0.127	−2.88	0.004	−0.62:−0.12	0.913/0.915
Rodents	Abundance	848	0.354	0.081	4.35	<0.001	0.19:0.51	0.263/0.897

## IV. DISCUSSION

We performed a systematic review and a field study to assess and synthesise the effects of protection nets on orchard‐dwelling biodiversity. Upon the retrieved evidence, we aim to disentangle the key mechanisms by which nets might impact the different levels of biological organisation (from individuals to the whole community), and whether such impacts may have undesired consequences for the supply of biodiversity‐mediated ecosystem services.

### (1) Review and case study

We found a relevant bulk of knowledge (48 studies, reporting 134 effects) on the impact of orchard netting on non‐crop biodiversity. Nevertheless, the available information is biased towards Western countries, apple orchards, and pest biota. This implies that many areas (e.g. Asia), crops (e.g. tropical fruits), and taxa (e.g. all vertebrates but birds) are significantly understudied, if not completely neglected.

The most striking gap is the complete absence of studies focusing on non‐crop plant communities under netted orchards. Net‐mediated floral changes are extremely likely, considering the nets' acknowledged effect on photosynthetic‐active radiation, microclimate and soil conditions (Mupambi *et al*., 2018). Such floral changes may have cascading impacts on ecosystemic functions like pollination (Campbell *et al*., 2017; García & Miñarro, 2014), pest control (Bugg & Waddington, 1994; Judt *et al*., 2023), nutrient cycling and water retention. Before our study, no mammal species had ever been assessed, despite many mammals being relevant for many orchard crops, both as pest controllers (e.g. insectivorous bats: Ancillotto *et al*., 2024) and pests (e.g. frugivorous bats, herbivore ungulates, primates and rodents; Marada *et al*., 2019; Mphethe *et al*., 2025; Suchomel *et al*., 2019).

Our first main finding is that protection nets exert a general negative effect on biota, for both pests and non‐pests. Yet, at times a few species were favoured by netting, increasing their abundance and/or incidence under the nets. For instance, in one of the very few mid‐term studies (5 years of sampling), Chouinard *et al*. (2017) recorded the gradual built‐up of a non‐target pest species (*Choristoneura rosaceana*) in apple orchards covered with exclusion nets (that successfully blocked target pests – *Cydia pomonella*, *Rhagoletis pomonella* and *Lygus lineolaris*). In our case study, anti‐hail nets favoured a pest group, rodents, while hampering the diversity and abundance of both birds and flower‐visiting insects. Hence, as a second main finding, nets can exert contrasting impacts on different species, resulting in modified biological communities under and/or nearby the nets. The consequences of such changes should thus be evaluated when assessing the sustainability of this protection measure.

#### (a) Effects on pests and diseases

Most studies focused on insect pests (e.g. *Cydia pomonella* for apple orchards, or *Drosophila* flies for stone fruits and grapes). From available articles, a broad consensus on the efficacy of protective (especially exclusion) nets in reducing pest abundance (and/or damages) could be drawn. Interestingly, netting systems seem to provide a double‐fold protection (i.e. both against pests and weather, irrespectively of the original net function): exclusion nets protected from weather hazards (Charlot & Weydert, 2017; Chouinard *et al*., 2017; Ebbenga, Burkness & Hutchison, 2019), while anti‐hail nets also controlled pest populations (Baiamonte *et al*., 2016; Candian *et al*., 2018; Lueje *et al*., 2024; Tasin *et al*., 2008). Similarly, exclusion nets designed for one taxon can also protect from other pest taxa: Alt'Carpo nets, designed against the codling moth *Cydia pomonella*, also prevented damages from rosy apple aphid *Dysaphis plantaginea* (Dib, Sauphanor & Capowiez, 2010), whereas anti‐*Drosophila* nets also lowered wasp and bird damages in wine grapes (Ebbenga *et al*., 2019).

The basic mechanism of exclusion nets (with their fine mesh and tight envelope) is to physically impede pests' access to the netted environment, acting as a physical barrier. Nevertheless, pest control was also performed by anti‐hail nets, despite their larger mesh size and their roof configuration (i.e. incomplete isolation; Fig. 1). This suggests other mechanisms than the simple physical barrier. Tasin *et al*. (2008) were the first to propose that the observed reduction of *Cydia pomonella* under anti‐hail nets could be due to a net‐induced behavioural disruption on moth males while performing mating flights.

Owing to their general effectiveness, exclusion nets have been often presented as an environmentally‐friendly alternative to pesticides for controlling pests (Nissen, George & Topp, 2005). For this reason, some studies compared netting with pesticide‐spraying treatments as two alternative control strategies (Candian *et al*., 2020; Ebbenga *et al*., 2019; Nelson, Meys & Hutchison, 2024), with nets usually out‐performing pesticides. As a means of pest control, exclusion nets do not suffer from induced pest resistance, as pesticides do; nevertheless, pests' behavioural adaptations to nets could eventually limit nets' efficacy over time (Nelson *et al*., 2024; Siegwart *et al*., 2012).

When tested in relation to fungal, bacterial or viral diseases (e.g. the apple scab *Venturia inaequalis*, Huanglongbing and the sooty blotch, respectively), neither insect‐pest‐designed exclusion nets nor anti‐hail nets showed good protection levels (Bogo *et al*., 2012a,b; Boutry *et al*., 2022; Holtz *et al*., 2020; Lueje *et al*., 2024). This could be due to the higher leaf surface humidity under the nets that can benefit these organisms. The adoption of anti‐rain nets proved to be more effective (Holtz *et al*., 2020; Schluchter *et al*., 2022), yet other diseases (e.g. powdery mildew) may take advantage of the new drier conditions (Boutry *et al*., 2022; Holtz *et al*., 2020). For those diseases that depend on insect vectors (e.g. Huanglongbing), exclusion nets might (*i*) control the disease by blocking the vector (as they block insect pests), despite the more suitable microclimatic conditions under the nets (Ferrarezi *et al*., 2019; Zhang *et al*., 2023a), or (*ii*) foster the disease by excluding the predators of the vector, that can then grow in numbers and spread the disease (Boutry *et al*., 2022).

In this study, protective nets were evaluated on a mammal (pest) group, rodents, for the first time. We found that anti‐hail roof nets do favour these animals, likely by protecting them from their main predators – i.e. diurnal and nocturnal raptors – whose predation is hampered due to the net cover (see Appendix S5 for study limitations).

#### (b) Effects on non‐pest taxa

Netting also affects non‐pest biota. Such impacts were investigated by studies that, for the vast majority, still focused on pest‐related taxa, especially arthropod natural enemies. Only a very few studies investigated taxa not explicitly related to agricultural production, yet still valuable for biodiversity conservation or ecosystem services (e.g. birds). Amphibians, reptiles, and (non‐pest) mammals have never been assessed so far. Protective nets lead to a reduction in the abundance and/or richness of non‐pest arthropods (Dib *et al*., 2010; Lefebvre *et al*., 2016; Meissle *et al*., 2023; Nelson *et al*., 2024; Pajač Živković *et al*., 2019, 2018). This involves both ground‐dwelling (e.g. beetles and spiders in Pajač Živković *et al*., 2018, 2019) and arboreal guilds (e.g. spiders or predatory mites in Dib *et al*., 2010; Lefebvre *et al*., 2016; Marsberg *et al*., 2024). The same ‘physical barrier’ acting on insect pests may act on similarly‐sized non‐pest organisms, resulting in their reduction under the nets. At times, along with a general reduction in arthropod abundance and richness, a few species significantly increased under the nets (Meissle *et al*., 2023), suggesting more complex processes, such as a reduction in predation pressure and/or competition.

All the studies investigating pollinators and non‐pest birds found a negative effect of protection nets on them. Notably, both these two taxa have been suggested as ecological indicators in farmed landscapes (Anderle *et al*., 2024; Dalpasso *et al*., 2025), which should warn about the potential cascading effects of netting on biological communities.

In our case study, netted orchards host less numerous and less diverse assemblages of flower‐visiting insects (as also reported by Meissle *et al*., 2023; and Corti *et al*., 2026; see also Granata *et al*., 2025). Considering that some of these flower visitors might be pest predators and/or parasitoids (Ollerton, 2017), their reduction might undermine pest control, along with pollination service. Anti‐hail roof nets might obstruct insects while flying into the parcel and/or increase their mortality as observed during our field study (they get fatally entrapped in the net mesh; *personal observations*; Fig. S8). Netted orchards adjacent to (semi‐)natural habitats might thus act as sinks, eventually affecting populations also at broader scales, especially for the most mobile taxa (e.g. Hymenoptera and Diptera).

We also observed a reduction in the abundance and the diversity of birds in the parcels covered with anti‐hail nets. Despite netted parcels potentially providing additional structures to be used as singing, nesting or hunting perches, or potentially offering new foraging opportunities (chaffinches *Fringilla coelebs* and Italian sparrows *Passer italiae* were occasionally seen foraging on the insects trapped into the net mesh; *personal observations*), birds avoid using netted orchards. The roof cover might represent a (real or perceived) barrier, hampering access or escape possibilities. Indeed, chasing raptors – goshawks *Astur gentilis* and sparrowhawks *Accipiter nisus* – were observed hunting for thrushes and smaller passerines under the nets, taking advantage of their reduced possibilities to escape (*personal observations*). The foraging suitability beneath nets may also be reduced following changes in ground‐dwelling fauna or in the plant community. Our results are consistent with those found (at a broader scale) by Brambilla *et al*. (2015, 2013) in the same region 12 years earlier, reporting lower densities of song thrush *Turdus philomelos* and chaffinch under higher cover of anti‐hail nets. Notably, most of the species we investigated have a largely insectivore diet when breeding, being potential controllers of arthropod pests (García *et al*., 2024); their reduction in abundance and diversity may thus undermine the amount and the stability of pest control services in apple orchards.

In summary, the general reduction in non‐pest organisms should be seen as a drawback of protection nets, considering (*i*) the importance of halting biodiversity loss in agroecosystems, and (*ii*) the important ecosystem functions supplied by many of these non‐pest species (most notably, pollination and pest control) that eventually underpin crop production.

#### (c) Effects on crop pollination

As a result of the above‐discussed impacts, protection nets can hinder crop pollination: enclosing the orchard during bloom can severely alter pollinators' activity, reducing fruit set or quality (Boutry *et al*., 2022; Elsysy *et al*., 2019). This can eventually impact crop yield and farmers' income (Bonneau *et al*., 2021; Corti *et al*., 2026). The adoption of protection (especially exclusion) nets thus implies a trade‐off between pollination and pest‐protection. When feasible, temporal adjustment in net usage reduces this trade‐off, as usually done for *Cydia* control, whose nets are activated after pollination has occurred. Nevertheless, some pests require a control during the crop pollination phase. In these circumstances, two strategies were found: (*i*) temporarily open the nets to allow pollinators to enter the orchard until reaching an acceptable amount of pollination time (~30 h in Chouinard *et al*., 2017), which inevitably exposes crops to pest colonisation; or (*ii*) place colonies of domestic pollinators (e.g. honey or bumble bees) inside the nets, which may lead to additional costs for the farmer (and for wild pollinators; Granata *et al*., 2025).

#### (d) Effects on pest control

Protection nets may also alter pest control, however their effects are less clear. For instance, Marsberg *et al*. (2024) found a significant increase in predatory arthropods and their pest‐control capacity in South African orange orchards where full enclosures were deployed. Conversely, more studies recorded significant reductions in beneficial arthropods under the nets (Boutry *et al*., 2022; Candian *et al*., 2021, 2020; Nelson, Klodd & Hutchison, 2023). Yet, netted environments witnessing reduced pest‐control potential might also benefit from reduced pest pressure (since pests are also impacted by netting). Whether the fewer pest controllers would be enough to control the fewer pests is likely context‐specific, and partly stochastic. Nevertheless, a detrimental disruption of the bio‐control dynamics has been observed by Marshall & Beers (2022), who recorded the increase of an aphid pest species inside exclusion nets despite the co‐occurrence of two natural enemy species. A temporal dynamic might modulate pest control: pest controllers/natural enemies, due to their larger size, might require more time to colonise the netted environment, hence reducing their chances to control pests while still occurring at low numbers (thus violating the ‘predator‐in‐first’ hypothesis; Gontijo, 2019).

Therefore, when devised against pests, protection nets should be intended as a non‐selective pest‐control measure, somewhat comparable to many broad‐spectrum pesticides. Disclosing these unintended effects of netting on relevant non‐pest taxa is key for long‐term sustainability in crop production and to design effective strategies for pest control.

Overall, these unintended effects on biota – and on the ecosystem functions that wild species provide – should be considered in sustainability evaluations of orchard netting. In our case study, anti‐hail nets promoted a pest group, rodents, while reducing ‘helpful’ animals like birds and flower‐visiting insects. By altering the supply of key ecosystem services, netting may increase farmers' dependence on pesticides, rodenticides, and on renting honeybees' colonies. The evaluation of the overall effectiveness of anti‐hail nets should therefore balance fruit protection from hailstorms with the potential drawbacks arising from net‐mediated biotic alterations.

### (2) The ‘netted ecosystem’ and its biological community

The biological effects of netting found both in the reviewed literature and in our case study may be interpreted as direct impacts of nets or as the indirect consequences of net‐induced environmental changes. Such effects may act individually, jointly or synergically, by e.g. altering available resources or the five ecosystemic features we highlighted in Section I (higher isolation; altered disturbance regimes; altered structural complexity; more marked temporal fluctuations; at times, reduced spatial extent). Here, we reframe the reviewed results into a multi‐level synthesis, according to three levels of increasing biological complexity (from the species to the community level). Despite our attempt to provide a holistic synthesis, we reaffirm that net types (i.e. exclusion *versus* weather‐protection nets) and net characteristics (structural configuration, mesh size, colour and activation timing) would eventually drive the ultimate impacts of netting (e.g. Chouinard *et al*., 2023; Fornasiero *et al*., 2023).

#### (a) Species level

**Fig. 5:**
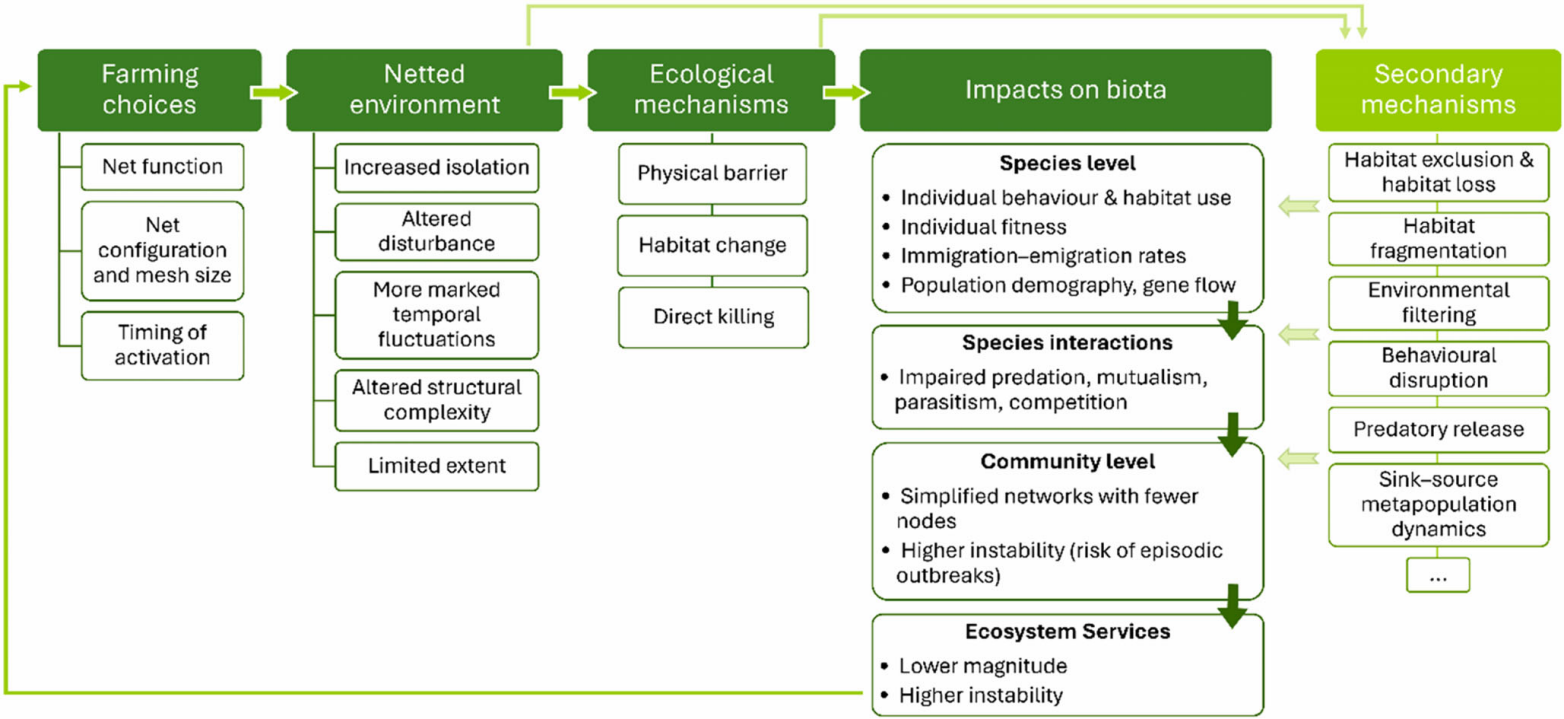
The proposed framework about the effects of orchard netting on biodiversity. Note that net‐induced changes on biota can have cascading effects on the supply of ecosystem services upon which farming choices are taken.

Nets can impact biota at the species level by acting as (*i*) a physical barrier, (*ii*) a modulator of habitat changes that individuals need to cope with, or (*iii*) a cause of direct killing (Fig. 5).

When acting as a physical barrier, nets impede or impair animal movements. Complete exclusion systems lead to demographic collapse inside the nets due to null immigration rates (as long as biota are not already present before net activation; Chouinard *et al*., 2023). Despite their non‐exclusion function, even nets for protection from adverse weather, like anti‐hail nets, may alter immigration rates and animal movements by physically obstructing individuals as they move through the netted system.

Once entered, organisms may find altered conditions therein, as nets may induce either biotic or abiotic changes in the orchard environment (the latter being largely reviewed by the agronomic literature). These changes affect the suitability of netted orchards to many species, leading to altered behaviour or fitness (Evans *et al*., 2019; Sauphanor *et al*., 2012). For instance, Nelson *et al*. (2024) suggested that anti‐hail nets hampered the flight navigation capacity in Anthocoridae, after a reduction in ultraviolet (UV) light beneath the nets (see also Candian *et al*., 2020). Net‐induced habitat changes might also come in the form of increased structural complexity that hampers individual behaviours, as suggested for anti‐hail net covers disrupting codling moths' mating flights (Sauphanor *et al*., 2012; Tasin *et al*., 2008) and passerine escape from chasing raptors (*personal observation*). When their adoption is followed by a reduction in pesticide treatments, nets might result in lower disturbance, allowing the partial recovery of arthropod communities formerly depleted by pesticides (Lefebvre *et al*., 2016; Marsberg *et al*., 2024).

Interestingly, no studies assessed nets as a cause of direct killing, however we found scattered evidence for this (potentially widespread) phenomenon. In our study area (Non Valley) we observed that flying arthropods and passerine birds can get lethally entrapped within the mesh of anti‐hail roof nets (Fig. S8). In Japan, citrus farmers were reported to use intentionally‐loosen nets to induce the accidental lethal trapping of the vulnerable frugivorous bat *Pteropus dasymallus* (Charerntantanakul, Shibata & Vincenot, 2023), considered a pest. Net‐induced killing may thus pose a direct threat to the conservation of the species.

Overall, we propose body size as the possible ultimate factor driving species‐level responses to netting. Indeed, on the one hand, the body/mesh size ratio sets the degree of permeability of the netted orchard for a species, with species larger than the mesh size being left out from exclusion nets or witnessing hampered accessibility to sites covered with other net types (e.g. anti‐hail nets). On the other hand, body size is linked to the species' use of space, which affects their possibility to come across the netted orchards within their home ranges or to develop viable populations just inside netted systems.

In general, larger (or more‐mobile) species may respond *behaviourally*, by adapting their foraging or habitat selection patterns (Bouvier *et al*., 2022; Mansouri *et al*., 2020), which may then propagate into altered fitness or survival and into altered population densities at broader‐scales (e.g. Brambilla *et al*., 2015, 2013; Lefebvre *et al*., 2016). Such behavioural adaptations (and the net barrier *per se*) may thus result in habitat loss and/or fragmentation for the species, though these aspects have never been investigated so far.

Conversely, species small enough to establish viable populations within a netted orchard (e.g. microbes or small arthropods) may be affected either *physiologically* or *demographically*. Depending on their capacity to pass through the nets and their sensitivity to net‐induced changes, nets may trigger altered immigration, emigration, mortality, and growth rates. Fungal diseases, for instance, were often observed spreading under the nets due to more favourable conditions thanks to increased leaf surface wetness (Bogo *et al*., 2012a).

After body size, other functional traits – firstly locomotion and habitat specialisation – may influence species' responses to netting, though additional evidence is needed. Flying species may be more affected by netting than terrestrial ones, as suggested by the evidence of direct killing (in flying arthropods, birds, and bats). Secondly, arboreal taxa may also be affected, especially by exclusion systems that envelop their primary habitat (i.e. the canopy): Lefebvre *et al*. (2016) observed significant changes in the occurrence of an arboreal spider in orchards that were enclosed in single‐row exclusion nets, while no effects were found for ground‐dwelling spiders in similar settings (Pajač Živković *et al*., 2019). Tree‐nesting birds are forced to find alternative nesting habitats after the adoption of exclusion nets (Bouvier *et al*., 2022); notably, even weather‐protection nets may discourage nesting behaviours, as observed in Moroccan orchards covered by anti‐hail roof nets – where no successful nesting by the turtle dove (*Streptopelia turtur*) was recorded (Mansouri *et al*., 2020).

Depending on the body size and other functional traits, the species‐level consequences of netting may involve either physiological, behavioural or demographic adaptations, that may easily propagate outside the netted system (Poinas *et al*., 2025) and to the other nodes of the network.

#### (b) Species interactions

As a cascading effect, the changes in individual behaviours and/or population demography mediated by the physical barrier, induced habitat changes, and direct killing, lead to new arrangements of the ecological interactions within the orchard community. This mechanism explains well the gradual build‐up of non‐target pests due to reduced predation, once the predators are left out from the netted environment (or altered in their hunting efficiency), as suggested for woolly apple aphids (*Eriosoma lanigerum*; Marshall & Beers, 2021), rodents (this study) and spiders (Lefebvre *et al*., 2016). Dib *et al*. (2010) suggested a disruption in the ant–aphid mutualistic relationship, with lowered aphid infestation due to fewer ants under the nets. Chouinard *et al*. (2023) linked the observed increase in an arthropod pest species (*Choristoneura rosaceana*) under the nets with a significant reduction in the parasitism rates they suffered during the overwintering generation. These three examples respectively suggest a disruption in predatory, mutualistic, and parasitic relationships within the ‘netted community’.

Observed reductions in bird densities in netted orchards might be driven by a reduction in foraging suitability (Brambilla *et al*., 2015, 2013; this study), which again implies the disruption in interspecific interactions, potentially involving pest‐ and weed‐control. Thrushes (like song thrush *Turdus philomelos* and common blackbird *T. merula*) and small passerines are likely to experience higher predation rates by hawks in netted environments, providing another example of altered predator–prey dynamics due to netting.

#### (c) Community level

Because of the aforementioned environmental features (increased isolation, altered disturbance, etc.) and ecological dynamics (species filtering, behavioural adaptation, and disruption of interactions), we can expect species‐poorer communities and simpler food‐webs under the nets. Although no study has ever assessed the ‘netted community’ as a whole, several studies have reported a decrease in guild richness or abundance (Boutry *et al*., 2022; Dib *et al*., 2010; Marshall & Beers, 2021; Meissle *et al*., 2023; Nelson *et al*., 2024; this study). Apex predators, due to their larger size, are most likely left out from netted orchards, which implies a reduction in the delivery of top‐down (pest) control. Such a community might be more exposed to the risk of episodic outbreaks or gradual build‐ups of taxa no longer controlled by predators (as reported in Alaphilippe *et al*., 2016; Chouinard *et al*., 2023; Gaire *et al*., 2022; Holtz *et al*., 2020; Lloyd *et al*., 2005; Marsberg *et al*., 2024; Marshall & Beers, 2022). Again, this outcome would be largely dependent on the net function/configuration and the management decisions that ultimately drive the degree of net‐induced habitat changes (Fig. 5). In turn, the reduction in both abundance and species richness of several taxa would lead to a reduction in, respectively, the magnitude and the stability of the ecosystem services supplied or mediated by those taxa (Shackelford *et al*., 2013).

Notably, by involving species of different body sizes and ecosystem processes acting at multiple spatio‐temporal scales (Wiens, 1989), most of the above mentioned dynamics exceed the netted environment itself, which calls for landscape (multi‐scale) approaches, rarely adopted so far (Brambilla *et al*., 2015, 2013; Lefebvre *et al*., 2016; Poinas *et al*., 2025). No study has attempted to quantify the spatial magnitude of the net‐induced ecological perturbation on any species, interaction or community. Yet, such information would greatly improve our understanding of the true impacts of netting at broader scales (see also Appendix S6 for additional considerations about the reviewed sampling designs).

### (3) Implications for biodiversity conservation

Ongoing agricultural intensification is leading to a spread of netting systems over an ever‐increasing extent of orchards. This trend might exacerbate current biodiversity loss in agroecosystems worldwide (Emmerson *et al*., 2016). From a conservation perspective, the most detrimental effects of orchard netting on wild taxa are probably habitat loss, degradation and fragmentation. Larger species, and especially flying apex predators (like birds and bats), may be strongly impacted due to nets impeding or altering movements and foraging behaviours (Ancillotto *et al*., 2024).

However, there is still a considerable knowledge gap to be filled, especially for plants and vertebrates, as for biota from lower latitudes. So far, human‐driven habitat modifications have favoured a small pool of more synanthropic/generalist species, reducing the diversity and resistance of natural communities (Etard, Pigot & Newbold, 2022; Karp *et al*., 2012; Rolls *et al*., 2023; Smart *et al*., 2006): a similar outcome may result from the widespread use of netting. Indeed, such a spread will further alter the orchard environment by increasing the number of artificial components, like poles and synthetic nets, thereby expanding human‐altered habitats. Only when associated with a sensible reduction in other detrimental impacts (e.g. agro‐chemicals), will the overall impact of netting on natural communities be lower (Marsberg *et al*., 2024); yet the habitat loss exerted by nets may still significantly impact several taxa.

### (4) Implications for orchard management

To fully evaluate orchard netting as a sustainable farming practice, several aspects neglected in the literature must be addressed. The most relevant gap to fill is the recognition of the interactive link between orchard netting and the several ecosystem services sustaining crop production. Specifically, it is crucial to understand the impact of nets on ground flora, wild pollinators, key pest controllers (e.g. insectivorous bats) and soil fauna. These taxa have been largely overlooked so far, despite their disproportionate role in supplying crop‐supporting ecosystem functions such as pollination, pest control and nutrient uptake (Granatstein & Sánchez, 2009; Judt *et al*., 2023; Maas *et al*., 2016; Mashilingi *et al*., 2022).

The occurrence of adjacent semi‐natural habitats might partially compensate for nets' negative effects on biodiversity (Bouvier *et al*., 2022), hence the acknowledged claim for a higher heterogeneity in farmlands (Priyadarshana *et al*., 2024) might hold true also for compensating the impacts of orchard netting. Future research should explore the interaction between agricultural practices (e.g. netting) and the surrounding landscape (Poinas *et al*., 2025; Saunders & Luck, 2018; Shackelford *et al*., 2013; Wang *et al*., 2022). This would provide evidence‐based guidance (and possibly incentivise top‐down regulations; Martinez‐Nuñez *et al*., 2025) for the spatial planning of orchard districts by the major crop companies.

Kelderer *et al*. (2018) also raised the question of the carbon footprint of protective nets, which has never been quantitatively assessed in our reviewed studies (but see Hofmann *et al*., 2023). Indeed, the usual claim of exclusion nets being the ‘environmentally‐friendly alternative to pesticides’ should be further explored. Although their effectiveness in pest control has been demonstrated (Candian *et al*., 2020; Ebbenga *et al*., 2019; Nelson *et al*., 2023; see also Chouinard *et al*., 2017) and may reduce pesticide applications, nets' actual sustainability depends also on other factors that were not addressed in the reviewed studies. By opting for netting instead of spraying, we are moving from the local impacts of pesticides to external impacts of net production and disposal. This dynamic of externalisation of the impacts is easily overlooked in current analyses and may depict netting as a more sustainable practice than it really is.

We support the point raised by Lueje *et al*. (2024) about the aesthetic consequences of the adoption of netting over large extents (also mentioned in Candian *et al*., 2018), which can have economic consequences in some traditional farming landscapes that also depend on tourism (e.g. the Non Valley or South Tyrol; Kelderer *et al*., 2018). Crop companies might also want aesthetically pleasing orchard landscapes to make their products more appealing to the growing segment of environmentally‐conscious consumers.

#### (a) Alternatives

While in a few specific cases netting may be the only practical option (according to Kelderer *et al*., 2018; e.g. for organic cherry plantations), for many crops and IPM systems, alternative crop‐protection strategies exist and should be assessed comparatively. Against meteorological hazards, an alternative (never reported in the reviewed studies) is the adoption of insurance policies, which could be more profitable to farmers under some circumstances (Porsch, Gandorfer & Bitsch, 2018; Rogna, Schamel & Weissensteiner, 2021). In case of damages, e.g. by hailstorms, farmers are compensated by the insurance agency for the lost income due to reduced crop value (although damaged fruits may still retain some profitability for processed products like juices). Adopting this ‘insurance strategy’ would prevent the use of nets (and all its consequences) but would inevitably increase the proportion of damaged fruit relative to marketable yield, potentially reducing farmers' competitiveness in the fresh produce markets.

For pest control, none of the reviewed studies mentioned, nor are we aware of, comparable insurance policies. Regardless, the integration of multiple control practices might reduce our dependence on both pesticides and netting, while still providing adequate levels of pest control. This might include augmenting releases of natural enemies (Dib *et al*., 2010; Marshall & Beers, 2021), pest mating disruption (Romet, Severac & Warlop, 2010), sterile insect techniques, and viral treatments (Shaw, Nagy & Fountain, 2021). Yet, such integrations have never been rigorously tested in relation to orchard biodiversity. In these and all future assessments, cost feasibility (Del Fava, Ioriatti & Melegaro, 2017; Digiacomo *et al*., 2023) and labour times for net installation and activation should also be accounted for (Johnson, Fortna & Manoukis, 2020; Schluchter *et al*., 2022). For instance, single‐panicle or single‐tree nets might be more labour intensive but may impact less on non‐target biota, especially when leaving the ground free, offering potential habitats to many species. To fully explore the profitability of different net configurations, future research should implement such comparisons, and context‐specific decision trees combining pros and cons of different netting options could be developed to guide decisions by planners and farmers.

Finally, current frontiers in agriculture are now exploring the use of photovoltaic‐powered structures that would enable remote activation of protection nets only when needed (e.g. during a hailstorm). This alternative is still under development and very few data are available (e.g. Karaman *et al*., 2023). However, we anticipate that this ‘technologically intense’ alternative may become feasible in the future, at least in some regions.

## V. CONCLUSIONS

(1) Protective nets are a widespread measure in orchard management worldwide, but their effect on biodiversity has been poorly addressed (van der Meer *et al*., 2020). By performing a global review and a dedicated field study, we aimed to fill this knowledge gap, highlight the ecological mechanisms involved, and identify the main avenues for future research.
(2) Available evidence is biased. Much of the literature deals with apple orchards, with a small fraction of studies focusing on tropical fruits. Moreover, organisms of high agronomic relevance (i.e. microbial diseases, insect pests and their natural enemies) have been disproportionately assessed, with other taxa (primarily, mammals, wild pollinators, and plants) being largely to completely ignored.
(3) When focusing on their specific purpose, all net types proved to be effective, at times offering a twofold protection (from both pests and weather hazards). Yet most of the literature neglected the indirect environmental impacts of netting. Indeed, when non‐target taxa were investigated, significant changes in their abundance, diversity and behaviour were found.
(4) We clarified the mechanisms by which nets can affect biota across levels and the intrinsic species traits that modulate their responses to netting. Protective nets act as physical barriers, as modulators of habitat changes, or as a cause of direct killing. Depending on the species' size and locomotion, this leads to changes in colonisation rates or individual behaviours, ultimately influencing population demography. Such species‐level changes lead to impaired interactions (i.e. predation, mutualism and parasitism), ultimately resulting in new arrangements of the whole community and in consequential changes in the ecosystem services and disservices mediated by such a community.
(5) The collected evidence suggests that netted orchards generally host poorer communities with weaker interactions, leaving out many predators. The resulting trophic networks are simplified and less stable, increasing the risk of pest outbreaks. To compensate for this reduced top‐down control, farmers may eventually necessitate greater pesticide use.
(6) By revealing such ecosystemic dynamics, we highlight the potential long‐term (indirect) consequences of adopting nets as a protective measure against pests and/or weather hazards. The several trade‐offs involving other ecosystem services mediated by biodiversity (i.e. pollination, pest control and nutrient uptake) may reduce the positive contribution of nets on crop production. We thus encourage future research to design long‐term, multi‐scale studies on (*i*) understudied taxa (especially those responsible for ecosystem services), (*ii*) landscape dynamics and netting–landscape interactions, and (*iii*) alternative practices (e.g. insurance policies) or the integration of current ones (e.g. mating disruption) for reaching sustainable crop protection.
(7) In conclusion, the overall sustainability and practicality of orchard netting should be evaluated, weighing its ecosystem‐level consequences, including impacts on wild species and the ecosystem functions they provide.

## Supporting information


**Fig. S1.** Photographs of different configurations of protection nets in orchards.
**Fig. S2.** Views of the study area.
**Fig. S3.** The location of the 63 plots.
**Fig. S4.** PRISMA diagram of the literature review procedure adopted in this study.
**Fig. S5.** Mesh size reported in the reviewed literature.
**Fig. S6.** Bar plots of the main topics explored by the reviewed literature.
**Fig. S7.** Effect sizes estimated by the full models for each of the three taxa investigated in the case study.
**Fig. S8.** Accidental fatalities of insect pollinators and birds due to anti‐hail roof nets in the apple orchards of the Non Valley, Italy.
**Table S1.** Dates of the surveys of the case study.
**Table S2.** List of the environmental variables collected for the case study, along with biological data on birds, flower‐visiting insects, and rodents.
**Table S3.** A summary of the most relevant information related to the three taxa investigated in the present case study.
**Appendix S1.** Technical information on protection nets.
**Appendix S2.** Detailed information on the sampling design of the case study.
**Appendix S3.** Detailed information on the statistical analyses of the case study.
**Appendix S4.** Detailed report of the results of the literature review.
**Appendix S5.** Limitations of the case study.
**Appendix S6.** Considerations on the sampling designs of the reviewed literature.
**Appendix S7.** Considerations on funding sources and conflicts of interest.

## Data Availability

Replication data are publicly available online in the repository of the University of Milan, at https://doi.org/10.13130/RD_UNIMI/06PWFK.
