# Supplementary material for: Orchard netting impacts on biodiversity leading to cascading effects at the ecosystem level
Source: Biol Rev Camb Philos Soc. 2026 May 19;101(5):2448–66. doi: 10.1002/brv.70186 (PMC13542310; doi:10.1002/brv.70186)
Supplement: Supplementary file 1 [file BRV-101-2448-s001.docx]

**Supporting information for “Orchard netting impacts on biodiversity leading to cascading effects at the ecosystem level”**

Corrado Alessandrini^†,*^, Karan Sethi^†^, Emanuela Granata, Ekaterina Mogilnaia, Valeria Vitangeli, Giovanni Zanfei, Chiara Fedrigotti, Paolo Biella, Dino Scaravelli, Luigi Marchesi, Péter Batáry, Diego Rubolini, Paolo Pedrini, Mattia Brambilla

^*^Author for correspondence (E-mail: [corr.alessandrini@gmail.com](mailto:corr.alessandrini@gmail.com); Tel.: +393469681323).

^†^Shared first authors

**Figure S1.** Photographs of different configurations of protection nets in orchards. Upper row: (A) single-canopy (exclusion) nets on young citrus trees in Florida, USA [Fig. 1 in Gaire *et al*. (2022)]; (B and C) single-row nets with ground also being excluded (namely, ‘complete exclusion nets’) in a Canadian apple orchard [Fig. 1 in Chouinard *et al*. (2017)] and a vineyard in Minnesota, USA [Fig. 1 in Ebbenga, Burkness & Hutchison (2019)]; (D) single-row (exclusion) nets in a Swiss cherry plantation additionally covered by anti-hail roof nets [Fig. 1C in Boutry *et al*., (2022)]. Middle row shows examples of roof nets: (E) roof (anti-hail/shade) nets in a vineyard in Peloponnese, Greece (early June 2025, photo by C.A.); (F) black and green photo-selective nets over apple trees in Shaanxi Province, China [Fig. 1A in Zhang *et al*. (2023*b*)]; (G) anti-hail nets stretched down to the ground, possibly to deter mammal herbivores (intensive apple orchards in Northern Hungary, March 2024, photo by C.A.). Lower row shows examples of full-enclosures: (H) in New Zealand kiwis [Fig. 1C in Evans *et al*. (2019)]; (I) an aerial view of a South African citrus orchard under full enclosure [Fig. 1 in Marsberg *et al*. (2024)]; (J) exclusion nets over a Hawaiian coffee plantation [Fig. 1 in Johnson, Fortna & Manoukis (2020)]; (K) inner view of a full-enclosure netting system in a Canadian apple orchard [Fig. 1 in Chouinard, Firlej & Cormier (2016)].

**Figure S2.** Views of the study area. (A) Map of the Non Valley, with orchard areas in green and anti-hail netted orchards in red. The elevation gradient is represented by shades of grey (white lowest, dark gray highest elevation; range 210–2600 m above sea level); isolines show increments of 125m (pale brown) and 500m (brown). Upper-left insets show the location of the Non Valley in Europe and in the Alpine Region; background image from Esri Shaded Relief. (B) Two views of the orchard landscape, dominated and not-dominated by active anti-hail nets (upper and lower picture, respectively). (C) Three exemplary apple parcels without, with inactive, and with active anti-hail nets.

 **Figure S3.** (A) The location of the 63 plots. (B) One of the 63 plots, with the many parcels found within it coloured by the occurrence of anti-hail nets. (C) From left to right, the abundance of birds, flower-visiting insects, and rodent holes in that same plot. Stronger colours denote higher abundances (i.e. number of bird individuals, flower-visiting insects, and rodent holes, respectively). Bird and flower-visiting insect data also included species richness (not shown).

**Figure S4**. PRISMA (preferred reporting items for systematic reviews and meta-analyses) diagram of the literature review procedure adopted in this study. The search string is coloured by the different topics involved in the review (namely, nets, biodiversity, orchards, and impacts).The search string in Scopus had ‘Pre/x’ instead of ‘NEAR/x’, as to follow Scopus engine requirements. The full list of the retrieved articles can be found in the online Replication Data (see Data Availability Statement).

**Figure S5.** Mesh size reported in the reviewed literature (48 studies, of which 40 reported mesh sizes). Mesh size is expressed as the mesh area (mm^2^) per each ‘net function × net configuration’ combination (the blocks): the mean ± S.D. of mesh area is reported both numerically (text) and graphically (block texture). In parenthesis, the number of study combinations that reported mesh size in that block. The “combined” net function includes either exclusion or anti-hail nets with photo-selective and/or anti-rain nets. “n.d.” net function (one study) and “n.d.” net configuration (two studies) not shown.

**Figure S6.** Bar plots of the main topics explored by the reviewed literature (*N* = 48 studies), namely net function and configuration (in red), crop species, management regime and orchard type (in green), and the biological taxon, the agri-functional group it belongs to, and which response variable was investigated (in blue).

**Figure S7.** Effect sizes estimated by the full models for each of the three taxa investigated in the case study, in the apple orchards of the Non Valley, Trentino (Italy). Effect size is expressed in standard deviation units (the bars representing the 95% confidence intervals); higher transparency indicates lower statistical significance (higher P). For birds and flower-visiting insects, both their abundance and diversity were modelled (and presented in red and blue, respectively). For variable meaning and model specifications, see Table S2 and S3.

**Figure S8.** Accidental fatalities of (A) insect pollinators (mostly honey bees) and (B) birds (two serins *Serinus serinus*) due to anti-hail roof nets in the apple orchards of the Non Valley, Italy. Multiple fatality events were observed for both flying insects (also including solitary bees and bumble bees) and birds (also including greenfinch *Chloris chloris*, goldfinch *Carduelis carduelis* and chaffinch *Fringilla coelebs*).

**Table S1**. Dates of the surveys of the case study in the apple orchards of the Non Valley, Trentino (Italy).

| **Taxon** | **Number of surveys  (i.e. visits)** | **Dates (records)** |
| --- | --- | --- |
| Birds | 3 (year 2023) | 24.04–06.05 (617 sampled parcels); 19.05–31.05 (617); 07.06–19.06 (617) |
| Flower-visiting insects | 5 (year 2023) | 24.04–06.05 (185 sampled points);  21.05–02.06 (183); 14.06–20.06 (184);  02.07–15.07 (177);  16.09–19.09 (178) |
| Rodents | 3 (year 2023) | 24.04–09.05 (391 sampled parcels); 21.05–02.06 (156); 20.06 (5) |
|  | 1 (year 2025) | 27.06–30.06 (313 sampled parcels) |

**Table S2.** List of the environmental variables collected for the case study in the apple orchards of the Non Valley, along with biological data on birds, flower-visiting insects, and rodents. All these variables were recorded/calculated per each apple parcel included in the analyses; distances from land cover (LC) classes are calculated from the closest parcel edge; topographic variables are weighted means within the parcel area. B = birds; FVI = flower-visiting insects; RB = rodent burrows. The focal factor of the analyses (anti-hail netting) is highlighted in bold. ‘x’ included in the full model; ‘*’ not included due to multicollinearity; ‘-’ not available for that taxon.

| **Variable** | **Description** | **Label** | **B** | **FVI** | **RB** |
| --- | --- | --- | --- | --- | --- |
| ***Topographic and landscape features*** | |  |  |  |  |
| Elevation | Elevation above sea level (m) | elev | x | x | x |
| Terrain wetness index | Potentiality of water accumulation of the landscape | TWI | - | - | x |
| Distance to woods | Minimum distance to Woods LC class (m) | dist_Woods | x | x | x |
| Distance to grasslands | Minimum distance to Grasslands LC class (m) (includes pastures, meadows, and arable lands) | dist_GRASS | x | x | x |
| Distance to urbanised areas | Minimum distance to Urban LC class (m) | dist_Urban | x | x | x |
|  | transformed by log(x+30) | dist_Urban_transf | x | x | x |
| ***Management variables*** | |  |  |  |  |
| Inter-row sward height | Mean height of the grass within two rows (cm) | gr_intra_H | - | - | x |
|  | transformed by log(x+10) | gr_intraH_transf | - | - | x |
| Intra-row bare ground | Percentage cover of bare ground along the row (%) | gr_intra_barecov | - | - | x |
| **Active anti-hail net** | **Presence (1) / Absence (0) of active anti-hail net over the parcel** | **hail_open** | **x** | **x** | **x** |
| Bark treatment | Presence (1) or absence (0) of bark protection (by plastic cover or painting) | anyBark_tr | - | - | x |
| Weeding | Presence (1) or absence (0) of traces of weeding along the rows | anyWeed | x | x | x |
| Hive | Presence (1) or absence (0) of beehives in the subplot | hive | - | x | - |
| Mowing regime | 0 = unmown; 1 = partially mown; 2 = mown | mowing_regime | - | x | - |
| Sward height | Mean sward height within the insect sampling subplot (cm) | sward_height | - | x | - |
| Number of flower species | Number of flowering species on the ground within the sampling subplot (#) | nFLOWER_sp | - | x | - |
| Soil tillage | 0 = no tillage; 1 = tillage of the intra-row | soil_tillage | - | x | - |
| ***Structural variables*** |  |  |  |  |  |
| Tree height | Mean tree height of apple trees (m) | treeH | x | - | * |
| Tree diameter | Mean tree diameter at breast-height (cm) | tree_diam | x | - | x |
|  | transformed by log(x+2) | tree_diam_transf | x | - | x |
| Intra-row distance | Mean distance between two consecutive trees along the row (cm) | dist_intra | x | x | x |
|  | transformed by log(x-20) | dist_intra_transf | x | x | x |
| Inter-row distance | Mean distance between two rows (m) | dist_inter | x | x | x |
| Non-spindle training | Apple trees trained in a Spindle (0) or non-Spindle (1) growth form (either guyot or bi-axis). | nonSpindel | x | - | x |
| ***Sampling and weather variables*** | |  |  |  |  |
| Duration | Duration of the bird sampling at each visit | duration | x | - | - |
| Daytime | Hour of the sampling | hour | x | x | - |
| Session | Ordinal number of the current visit to the plot (1:5) | session | x | x | - |
| DOY | Day of the Year of the sampling | doy | - | - | x |
| Temperature | Temperature (°C) | temp | - | * | - |
| Wind | 0 = calm; 1 = weak; 2 = moderate | wind | - | x | - |
| Cloud | Percentage of cloud cover (%) | cloud | - | x | - |
| Distance from observer | Distance between the central line of the plot and the parcel centroid (m) | dist_to_line | x | - | - |
| Parcel size | Area of the parcel overlapping with the plot area (m^2^) | parc_size | x | - | - |

**Table S3.** A summary of the most relevant information related to the three taxa investigated in the present case study on the impact of anti-hail netting in apple orchards of the Non Valley (Trentino, Italy). All analyses were performed using Generalised Linear Mixed-Effect Models (GLMMs); “(1|…)” denotes random intercept models. For variable names and transformations, see Table S2. *for the exact dates of sampling, see Table S1. † = calculated at the ‘parcel’ × ‘visit’ level (i.e. 2310 records). ‡ = some subplots fell within the same parcel, but never during the same visit nor in the exact same point, hence these are not to be considered as revisits.

| **Taxon** | **Response** | **Temporal range*** | **Sample size** | **Sampling unit** | **No. of sampled parcels** | **No. of visits per parcel** | **Prevalence of active nets** | **Full model** | | |
| --- | --- | --- | --- | --- | --- | --- | --- | --- | --- | --- |
|  |  |  |  |  |  |  |  | **Fixed factors** | **Random factors** | **Other terms** |
| Birds | Abundance of birds per parcel per visit | Apr–Jun 2023 | 2310 | Parcel × visit | 770 | 3 | 11.1%† | hail_open + dist_inter + dist_intra + treeH + tree_diam + nonSpindel + anyWeed + elev + dist_Urban + dist_Grass + dist_Woods + duration + time_start + dist_to_line + session | (1\|Plot ID) + (1\|Parcel ID) | offset: log(parcel_area)  zi-form: dist_intra |
|  | Richness of bird species per parcel | Apr–Jun 2023 | 770 | Parcel | 770 | 3 | 17.9% | hail_open + dist_inter + dist_intra + treeH + tree_diam + nonSpindel + anyWeed + elev + dist_Urban + dist_Grass + dist_Woods + duration + time_start + dist_to_line | (1\|Plot ID) | offset: log(parcel_area) |
| Flower-visiting insects | Abundance of flower-visiting insects found on ground flowers | Apr–Sept 2023 | 907 | Sampling subplot | 740 | 1‡ | 13.6% | hail_open + dist_inter + dist_intra_transf + mowing_regime + soil_tillage + hive + nFLOWER_sp + flowers + sward_height + elev + dist_Urban_transf + dist_Woods + dist_Grass + hour + wind + cloud | (1\|session) |  |
|  | Richness of flower-visiting insect taxa found on ground flowers | Apr–Sept 2023 | 907 | Sampling subplot | 740 | 1‡ | 13.6% | hail_open + dist_inter + dist_intra_transf + mowing_regime + soil_tillage + hive + nFLOWER_sp + flowers + sward_height + elev + dist_Urban_transf + dist_Woods + dist_Grass + hour + wind + cloud | (1\|session) |  |
| Rodents | Number of rodent burrows in 40 m of tree-rows per parcel | Apr–Aug 2023  Jun 2025 | 848 | Parcel | 848 | 1 | 13.2% | hail_open + dist_inter + dist_intra_transf + tree_diam_transf + nonSpindel + anyBark_tr + gr_intraH_transf + gr_intra_bare + anyWeed + elev + TWI + dist_Urban_transf + dist_Grass + dist_Woods + doy | (1\|Plot ID) + (1\|year) | zi-formula: gr_intraH_transf |

## **Appendix S1 – Technical information on protection nets**

The major features of protective nets are mesh size, colour and shading factor. Mesh size refers to the size of the squared (triangular, or rectangular) grid. Mesh sizes below 3.1 mm are used for anti-insect (exclusion) nets, from 1.7 to 7.0 mm for shade/photo-selective nets, while from 2.5 to 4.0 mm are used for anti‐hail nets. Colour is induced by adding additives to the fibre, hence it affects the net durability. Black, green or transparent nets are the most widely used. By reflecting specific wavelengths, the colour of the net alters the photosynthetically active radiation (PAR) reaching the crop, which affects plant physiology and phenology. When intentionally deployed for inducing specific plant responses (e.g. dwarfism, branch production, phenological delays), coloured nets are called photo-selective nets (Shahak, 2014). Shading factor is the amount of light that a net blocks, and it depends on both colour and mesh size. The higher the shading factor, the lower is the intensity of the light that reaches the crop. Higher values are typical of shade nets, that are particularly used at lower latitudes, where sunburn is more likely. Nevertheless, even nets designed for other purposes (e.g. anti-insect) might significantly shade the crop, with potential consequences on the quality of the fruit (Roussos *et al*., 2022). As reported in Chouinard *et al*. (2016), net durability ranges from six to ten years under field conditions (Rigden, 2008; Sauphanor *et al*., 2012).

## **Appendix S2 – Detailed information on the sampling design of the case study**

The study area of the present study case is the Non Valley (Trento Province, Italy), centroid coordinates: 46°20'47.8"N 11°03'51.8"E. Our sample covered 8.1% of all apple orchards of the Non Valley; anti-hail nets covered 20.4% of the orchard extent we sampled.

Bird communities were surveyed by means of plot counts, i.e. by slowly walking along the 200m-long plot line and recording the precise location of any bird observed or heard within the plot area (i.e. 100m-wide buffer from the plot line). We performed three visits per plot during the breeding season (late April to mid-June, Table S1), from dawn to noon, to match the time during which most bird species are most active. The overall number of birds *seen* within each apple parcel overlapping the plot was then calculated per each visit (Fig. S3C): birds both under or above the nets were counted together, as both interacted with the netted environment; acoustic contacts were discarded since no precise location could be inferred. Within this orchard district, during the breeding season, no bird species cause any damage to the apple crops, hence all bird species detected can be considered as non-pest organisms. Moreover, many of them are insectivorous or granivorous species, whose diet includes apple pests (García *et al*., 2024). For this reason, we will consider the higher abundance of birds (even if irrespectively of the species) as a proxy for a potential increase in the supply of pest-control services.

Insect communities were investigated by visual counts at 3 distinct *sub-plots* of 2.5 m-radius randomly placed within the apple orchards of each plot at each visit (5 visits per plot, *N* = 945 sampling sub-plots; each sub-plot being separated by at least 50 m from the other sub-plots of the same visit, to reduce the risk of double counting the same individuals). The 5-minute sampling consisted of recording any insect visiting a flower on the ground or on the (apple) canopy, and identifying it at a broad taxonomic level (as in Granata *et al*., 2025): namely, honeybees (*Apis mellifera*), bumblebees (*Bombus* spp.), wild bees (Apoidea), wasps and ants (Vespoidea), syrphid flies (Syrphidae), other flies (Diptera), butterflies (Lepidoptera), true bugs (Rhynchota), beetles (Coleoptera), grasshoppers (Orthoptera), and ‘other insects’. Despite their different ecology and contribution to supporting ecosystem services/disservices, all these taxa are involved in ground flower and/or apple pollination, some of them also being predator or parasitoid species. The overall number of insects visiting *ground* flowers at each sub-plot was then calculated and associated with the parcel characteristics it was in. We chose not to include those insects visiting *apple* flowers since apple blooming largely (but not exclusively) occurred before net opening; on the contrary, flowering of the ground layer was more widespread during the whole season (i.e. both before and after net opening). For a more complete sampling description, see Granata *et al*. (2025), where a larger dataset encompassing a broader agricultural–natural gradient was investigated. Conversely, here we focussed only on orchard-dominated conditions.

Rodent densities were investigated by counting the number of burrows (Šálek *et al*., 2025) found along the tree rows, over a standardized distance of 40 m per parcel, in as many parcels per plot as possible (mean ± S.D.: 10 ± 4 parcels per plot; *N* = 848 parcels overall). The most common rodent species occurring in the area within and among orchards, based on a non-lethal trapping we performed in 2023 (*unpublished data*), are wood mouse (*Apodemus sylvaticus*), common vole (*Microtus arvalis*), and bank vole (*Clethrionomys glareolus*). All these species occur in apple orchards and can feed on the rooting system of fruit trees (Balciauskas, Balciauskiene & Stirke, 2019; Suchomel *et al*., 2019). Due to their similar body size and ecology, distinguishing them at the species level by the burrow characteristics was considered unfeasible, hence we hereafter refer to rodent densities (irrespectively of the species). Rodent burrows usually collapse within a few weeks after abandonment, hence their occurrence can be a valid proxy for rodent presence within a short spatio-temporal range. Notably, single dens may have more entrances, so that the apparent number of burrows may not coincide with the actual number of dens (or rodents). However, such an effect should be randomly distributed across netted and non-netted orchards, so it may well represent a valid proxy for the responses of rodent abundances to netting in the orchards (see also Šálek *et al*., 2025).

Sampling was carried out between late April and early September 2023 and in June 2025 (Table S1); the above-mentioned trapping was carried out during the April–Sept 2023 on a subset of the 63 plots, but we are confident this did not affect the abundance of rodent burrows analysed for this study (also considering the very low capturability rates we observed).

All environmental variables were collected parcel-wise (i.e. separately per each parcel overlapping the plot). Along with anti-hail nets’ occurrence and status, other structural and management variables included tree height, tree diameter at breast height (as a proxy of parcel age), inter-row and intra-row distance, the occurrence of weeding, height of the grass under the row and within the rows. Management (i.e. temporally dynamic) variables were recorded at each visit, while structural (i.e. static) variables were recorded once for the whole season (Table S2). Moreover, topographic and land-cover variables were derived from Geographic Information Systems (QGIS software v3.34.5; QGIS Development Team, 2024) using two high-resolution layers: the TINITALY Digital Elevation Model at 10 m/pixel resolution (Tarquini *et al*., 2023), and the EUSALP Land Cover Map (Marsoner *et al*., 2023). Finally, variables related to sampling and meteorological conditions were also recorded during each visit. See Table S2 for a complete list of recorded variables.

During fieldwork, we mapped the totality of apple parcels overlapping the 63 plots (*N* = 2153 parcels). Nevertheless, due to logistical constraints, we did not manage to record all the environmental parameters for each of these 2153 parcels. This led to using fewer parcels in the final models: i.e. 770, 740, and 848 apple parcels for birds, flower-visiting insects, and rodent burrow analyses, respectively. Among these parcels, the prevalence of active anti-hail nets was 17.9%, 13.6%, and 13.2%, respectively (Table S3).

## **Appendix S3 – Detailed information on the statistical analyses of the case study**

To control for different parcel sizes, bird data (either abundance or richness) per parcel was leveraged using parcel area as an offset. Bird abundances were assessed at each visit (i.e. sampling unit: parcel × visit), while bird species richness was assessed as the overall number of species found in each parcel across the three visits (i.e. merging the three visits into a single value of species richness per parcel; sampling unit: parcel). We are aware that parcels vary in size, position and arrangement relative to the plot line walked by the observer, which might introduce a bias (due to different bird detectability). Nevertheless, these differences between the parcels are: (1) controlled by the use of the random factor (Parcel ID), the offset [log(parcel_size)], and the covariate ‘distance from observer’ (dist_to_line); and (2) are constant across the three visits, hence they should not bias the estimation of the effect of active anti-hail nets on bird abundances. A zero-inflation formula was deemed necessary [and supported in terms of Akaike’s information criterion (AIC)], and was included in the full model as follows: ‘ziformula: ~ dist_intra’; expecting that the tighter the tree rows of the parcel, the more probable the occurrence of a false-zero, due to a reduction in the visibility through the parcel.

For the flower-visiting insects, the model with both Plot ID and sampling session as random factors was less supported (higher AIC) than the one with the sampling session only. We thus opted to retain the latter, since (1) the variances associated with the Plot ID random effect were almost null, and (2) no problems of spatial autocorrelation arose when Plot ID was removed.

Variance in the detectability of the target organism can introduce severe biases in regression analyses. While bird and pollinator data were less sensitive to this issue, rodent data suffered from the variability of ground conditions along the sampled track across different parcels (i.e. sampling units); despite our accurate search, the amount and the height of the grass along the tree rows interfered with the detection of the ground burrows: short grass or bare ground conditions increased burrow detectability, while dense and high vegetation decreased it. This issue could eventually lead to false zeros (recording zero burrows while they were actually present, but not detected) and lower estimates of burrow abundance (some burrows being missed due to their reduced detectability). To account for this variability in burrow detectability, the mean grass height under the trees (‘gr_intraH_transf’ variable) was included both in the ‘zero-inflation formula’ (the ‘ziformula’ argument in function ‘glmmTMB’) and in the ‘conditional formula’, to account for increased false-zeros and reduced numbers of rodent burrows, respectively.

All estimated models were run by means of the glmmTMB function of the homonymous R-package (Brooks *et al*., 2017); Negative Binomial error distributions were implemented using the ‘nbinom2’ family, therein. All continuous variables included in the conditional and zero-inflation formulas were centered and scaled before modelling (i.e. mean = 0 and S.D. = 1), to obtain effect sizes as variations in standard deviation units. Analyses of the model residuals were performed by means of dedicated functions in R packages ‘performance’ (Lüdecke *et al*., 2021) and ‘DHARMa’ (Hartig, 2024), and reported in the Excel Tables (Excel 2) and in the .pdf files provided as Supplementary Materials.

Analyses were run on R software (v 4.4.0; R Development Core Team, 2020), by means of the RStudio interface (v. 2023.09.0-463; RStudio Team, 2020) and the following R-packages: ‘glmmTMB’, ‘MuMIn’ (Bartoń, 2026), ‘sf’ (Pebesma, 2018), ‘performance’, ‘DHARMa’, ‘tidyverse’ (Wickham *et al*., 2019). Statistical significance was considered as *P* < 0.05.

## **Appendix S4 – Detailed report of the results of the literature review**

We believe terminology to be a primary aspect in science. Hence, here we report how protection nets have been termed in the 48 reviewed studies (see also the dedicated column in the Excel 1, hosting all the relevant information retrieved in each reviewed study).

Firstly, some authors used the term “screen” (Zhang *et al*., 2023*a*), “tunnel” (Chouinard *et al*., 2017), “cage” (Marshall & Beers, 2022), “cover” (Gaire *et al*., 2022), or “screenhouse” (Ferrarezi *et al*., 2019) rather than “net”, while flexibility was found in the use of “protection” and “protective” attributes for nets. Moreover, (1) exclusion nets were also named “exclusion cages”, “single-row exclusion nets”, “complete exclusion nets”, “insect-proof nets”; (2) anti-hail nets were also simply termed ‘hail nets’ (Bogo *et al*., 2012*b*,*a*; Kelderer *et al*., 2018; Mansouri *et al*., 2020), which we believe to be an incorrect yet attractive name for them. Some vague terminologies make it hardly possible to clearly identify net type or configuration, as for “non-permanent nets” (Stander *et al*., 2019), “individual protective covers” (Gaire *et al*., 2022), or simply “nets” (Charlot & Weydert, 2017).

Within the reviewed literature (*N* = 48 studies), the net types investigated were: exclusion nets (*N* = 29), anti-hail nets (*N* = 20), shade nets (*N* = 7, mainly named “photo-selective nets”), rain-proof nets (*N* = 4), multi-type nets (i.e. a combination of two net types, *N* = 2); while in 2 studies no information about the net type could be retrieved. The most studied net configurations were single-row nets [*N* = 30 studies, of with 9 focussed on ground-excluding “complete” nets, *sensu* Chouinard *et al*. (2016)], followed by roof nets (*N* = 13), and full enclosures (*N* = 9), with only two studies focussing on single-tree nets, and no studies on single-panicle nets (Fig. S6). The studied crops were apple (*N* = 35 studies), citrus *s.l.* (*N* = 5, including mandarins and oranges), wine grapes (*N* = 4), cherry (*N* = 3), peach/nectarine (*N* = 3), pear (*N* = 2), kiwi (*N* = 1), coffee (*N* = 1), and apricot (*N* = 1). Studies came mainly from the Northern Hemisphere (26 from Europe, 9 from USA, 2 from Canada, 1 from each of Morocco, Lebanon, and China), with only 6 studies from the Southern Hemisphere (2 from Brazil and from South Africa, 1 from Australia and from New Zealand; Fig. 3). Eighteen (18), 12 and 6 studies were carried out - respectively - on IPM, organic, and conventional orchards, with the remaining 18 studies that did not provide any information about management (Fig. S6). Mesh sizes were reported in 40 studies (83%), ranging from very tight meshes (0.3 × 0.3 mm, in full-enclosure exclusion nets in Ferrarezi *et al*., 2019) up to 8 × 4 mm (in roof anti-hail nets in Bogo *et al*., 2012*b*,*a*); only one study explored non-gridded meshes (triangular-shaped single-row exclusion nets in Marshall & Beers, 2022). More than 75% of the studies (*N* = 37) assessed the significance of the observed variations by means of a test statistic.

Notably, two thirds of the reviewed studies (32 out of 48) have been published in the last 7 years (since 2018), with only 2 studies published before 2010. Eight out of 48 studies were proceeding papers (7 from “*Proceedings of the International Conference on Organic Fruit-Growing*”, 1 from “*IOBC/WPRS Bulletin*”), while 27 studies were published in peer-reviewed journals with a Q1 Scimago ranking in 2023.

## **Appendix S5 – Limitations of the case study**

We investigated bird assemblages as a whole and not by species. We chose to do so considering that (1) we wanted to highlight the more general mechanism driving avian response to netting, which might go beyond species-specific aspects, and (2) no orchard-dwelling species is considered an apple pest, so all the species provide potentially positive (and possibly complementary) contributions to the farming system. For these reasons, we specifically focussed on the overall abundance and species richness of birds, as the best proxy to the potential contribution to the supply of ecosystem services. Regardless, modelling the drivers of abundances of single species or specific functional groups may provide further insights into potential avian services in relation to orchard netting.

We investigated flower-visiting insects but did not quantify flowers’ abundance within the plot, hence we cannot derive proper flower visitation rates from our data: this means that the fewer insects we recorded under the nets could come either from fewer flowers (visited at the same rate as in unnetted plots) or from fewer insects visiting the same amount of flowers (which means lower visitation rates), or a combination of the two. Considering the knowledge gap relative to the abundance/richness of plant taxa under the nets, we have no clues as to whether the former might be more likely than the latter. However, the high number of pollinators (bees in particular) stuck in nets, and the very likely access constraints imposed by nets, suggest that reduced visitation rates due to precluded access may play a key role. Nevertheless, whatever the main reason, the final output is that netted orchards host less flower-visiting insects, from which we can hypothesise that there is reduced pollination and pest-control potential.

We interpreted the observed increase in rodent burrows as a consequence of a predatory release mechanism due to impaired predation of aerial predators on rodents. However, it is also possible that burrows under the nets might last longer due to the increased protection from the weather (hence misleadingly inflating burrow densities under the nets): nevertheless, this possible bias is negligible considering the extremely high ground disturbance due to mowing and tractor use regularly taking place both in netted and unnetted parcels.

## **Appendix S6 – Considerations on the sampling designs of the reviewed literature**

The great majority of studies carried out a control-treatment design, where a netted orchard (the treatment) was compared with an unnetted one (the control). This treatment-control approach was particularly common in agronomic studies focussing on the incidence of pest taxa. These agronomic studies were carried out in semi-experimental conditions where nets were newly deployed over part of a non-commercial orchard (often covering only very few trees; e.g. nine in Pajač Živković *et al*., 2018, 2016; or twelve in Aoun & Manja, 2020), usually for one or two growing seasons only. Such semi-experimental approaches have the advantage of better controlling the experiment by comparing close-by conditions and could be well suited for investigations of abiotic factors or crop physiology, that more quickly respond to the netting. However, when working with biological communities that fluctuate and homeorhetically organise in space and time, such approaches might fail in ‘capturing the whole picture’, reproducing realistic management conditions, the actual extent of net covers, and their effect after years of real usage (acknowledged only by Chouinard *et al*., 2023). Moreover, biased estimates might also arise if the control and the treatment units are too close to each other (Lueje, Jácome & Servia, 2024; Poinas *et al*., 2025). Conversely, observational approaches studying commercial orchards might be more suited for ecological investigations, yet they require a more careful design and more complex analyses to retrieve unbiased estimates of the ‘treatment’ effect. Particular attention should be paid in the selection of the sampling units, so to embrace all the environmental gradients found in the study area, without shortfalls such as multicollinearity among the treatment factor (i.e. netting) and other covariates. Despite a generally bigger sampling effort, such observational studies have the great advantage of better recreating real-world conditions, also allowing more realistic comparisons of alternative farming strategies (when found in the study area; e.g. Bouvier, Boivin & Lavigne, 2022; Marliac *et al*., 2016, 2015). No proper before–after control–impact (BACI) design was ever applied within the reviewed literature: while many studies performed a control–impact assessment, none ever investigated natural communities before and after the adoption (or the activation) of protective nets.

Other study designs included (1) multiple sampling plots within the netted orchard (as in Zhang *et al*., 2023*a*), which helps to disentangle within-orchard variations (Hall *et al*., 2020), eventually leading to uneven pest-exposure (Zhang *et al*., 2023*a*) or uneven supply of relevant ecosystem services (e.g. pollination; Kendall *et al*., 2021; Mateos-Fierro *et al*., 2024) under netted systems; and (2) studies carried out at broader scales, covering a gradient of net cover over the landscape (as in Brambilla *et al*., 2015, 2013; Poinas *et al*., 2025).

Among the main features characterising netted orchards and described in the main text (isolation, disturbance, size, etc.), the size of the netted orchard has never been investigated so far, and neither has the spatial scale of nets’ effect on biota. Overall, a general lack of consideration for landscape-scale dynamics emerged: only very few studies accounted for landscape composition/configuration around studied orchards, or for the occurrence of relevant semi-natural habitats close-by (Bouvier *et al*., 2022; Brambilla *et al*., 2015; Lefebvre *et al*., 2016; this study). Given the ever-increasing availability of open-source satellite-derived data (e.g. land-cover maps and digital elevation models) to map such features, controlling for their effect has become more straightforward. The regression-based modelling framework proposed in our study case is capable of accounting for landscape’s effect (e.g. distance to woodland, or the overall Shannon diversity index of the landscape) while testing the effect of a ‘treatment’ factor (in our case, the occurrence of active anti-hail nets). Moreover, the methodology applied by Poinas *et al*. (2025) is particularly relevant to testing the interaction between the netting factor and other landscape variables that might mitigate (or exacerbate) the effects of netting.

A wider (multi-scale) sampling including surrounding habitats at different distances from the focal netted orchard would ultimately allow a quantification of the spatial magnitude of the ecological perturbation created by the netting system on biota: as explored in the main text, source–sink dynamics or behavioural adaptations might propagate outside the netted orchard, eventually impacting individuals, populations, and communities far from it. “*How far from it?*” could thus be a relevant question for future research. Moreover, additive effects might also occur so that the large-scale adoption of netting may trigger effects different from (and possibly stronger than) those observed at the orchard-scale (as investigated by most of the reviewed studies). Filling these knowledge gaps by a multi-scale sampling design would be pivotal to better predict the effect of the current spread of netting systems over entire orchard landscapes.

## **Appendix S7 - Considerations on funding sources and conflicts of interest**

Funding information in science is becoming more transparent. In the reviewed studies, most of the declared funding comes from public institutions, like the European Commission, National authorities, or Universities. Several studies reported to be - at least partially - funded by the crop industry (directly by the companies, or by associations/consortia run by them, as the present study is); yet very few acknowledge potential conflict of interests due to such financial support (e.g. Sonter *et al*., 2024). For this reason, we warn about the potential risk for an over-optimistic attitude towards the effectiveness of protective nets, due to the crop industry's interest in adopting such practice. Public funding should be guaranteed for developing truly independent research on this topic.
